# The C-Circle Biomarker Is Secreted by Alternative-Lengthening-of-Telomeres Positive Cancer Cells inside Exosomes and Provides a Blood-Based Diagnostic for ALT Activity

**DOI:** 10.3390/cancers13215369

**Published:** 2021-10-26

**Authors:** Yuan-Yin Chen, Rebecca Dagg, Yuchen Zhang, Joyce H. Y. Lee, Robert Lu, Nancy Martin La Rotta, Sandra Sampl, Medina Korkut-Demirbaş, Klaus Holzmann, Loretta M. S. Lau, Roger R. Reddel, Jeremy D. Henson

**Affiliations:** 1 Prince of Wales Clinical School, University of NSW, UNSW, Sydney 2052, Australia; yuan-yin.chen@unsw.edu.au (Y.-Y.C.); yuchen.zhang@uq.net.au (Y.Z.); rolu6008@uni.sydney.edu.au (R.L.); n.martinlarotta@unsw.edu.au (N.M.L.R.); 2 Children’s Cancer Research Unit, The Children’s Hospital at Westmead, Faculty of Medicine and Health, University of Sydney, Westmead 2145, Australia; rdagg72@gmail.com (R.D.); loretta.lau@health.nsw.gov.au (L.M.S.L.); 3 Children’s Medical Research Institute, Faculty of Medicine and Health, The University of Sydney, Westmead 2145, Australia; joyce.918@gmail.com (J.H.Y.L.); rreddel@cmri.org.au (R.R.R.); 4 Comprehensive Cancer Center, Institute of Cancer Research, Department of Medicine I, Medical University of Vienna, 1090 Vienna, Austria; sandrasampl412@gmail.com (S.S.); medina.korkut@ist.ac.at (M.K.-D.); klaus.holzmann@meduniwien.ac.at (K.H.)

**Keywords:** C-Circle Assay, ALT, telomere, telomere maintenance mechanism, exosomes, cancer diagnostic

## Abstract

**Simple Summary:**

A clinical test for alternative-lengthening-of-telomeres (ALT) could assist with cancer diagnosis and monitoring of disease progression. ALT-targeted anticancer treatments are being developed; however, there is no appropriate companion ALT diagnostic. The C-Circle biomarker is the only known ALT specific molecule and the C-Circle Assay the only quantitative ALT assay that is amenable to clinical use. We show here that C-Circles are secreted by ALT+ cancer cell lines inside the exosomes and are protected from nucleases. We also show that secreted C-Circles, like intracellular C-Circles, are an ALT-specific biomarker, and in high-risk neuroblastoma, the blood-based C-Circle Assay has the potential to be an accurate diagnostic for ALT cancer activity. Therefore, the secretion of C-Circles by ALT+ cancer cells in the exosomes provides a stable blood-based biomarker and, potentially, a clinical diagnostic for ALT activity, which is required for the development of ALT-targeted therapies as well as for the diagnosis and monitoring of ALT+ cancer.

**Abstract:**

C-Circles, self-primed telomeric C-strand templates for rolling circle amplification, are the only known alternative-lengthening-of-telomeres (ALT)-specific molecule. However, little is known about the biology of C-Circles and if they may be clinically useful. Here we show that C-Circles are secreted by ALT+ cancer cells inside exosomes, and that a blood-based C-Circle Assay (CCA) can provide an accurate diagnostic for ALT activity. Extracellular vesicles were isolated by differential centrifugation from the growth media of lung adenocarcinoma, glioblastoma, neuroblastoma, osteosarcoma, and soft tissue sarcoma cell lines, and C-Circles were detected in the exosome fraction from all eleven ALT+ cancer cell lines and not in any extracellular fraction from the eight matching telomerase positive cancer cell lines or the normal fibroblast strain. The existence of C-Circles in ALT+ exosomes was confirmed with exosomes isolated by iodixanol gradient separation and CD81-immunoprecipitation, and C-Circles in the exosomes were protected from nucleases. On average, 0.4% of the total ALT+ intracellular C-Circles were secreted in the exosomes every 24 h. Comparing the serum-based and tumor-based CCAs in 35 high risk neuroblastoma patients divided randomly into ALT+ threshold derivation and validation groups, we found the serum-based CCA to have 100% sensitivity (6/6), 70% specificity (7/10), and 81% concordance (13/16). We conclude that the secretion of C-Circles by ALT+ cancer cells in the exosomes provides a stable blood-based biomarker and a potential clinical diagnostic for ALT activity.

## 1. Introduction

Telomere maintenance mechanisms (TMMs), telomerase, and alternative-lengthening-of-telomeres (ALT), are hallmarks of cancer that are required by cancer cells for continued growth and survival [1], and provide diagnostic and therapeutic opportunities. ALT is used by approximately 10% of all cancers and is more common in those of a neuroepithelial and mesenchymal origin, in some types of which it is the predominant TMM and is a prognostic indicator [2]. Unlike telomerase, ALT is not associated with the expression of a unique enzyme activity, and the identification and quantitation of the ALT activity depends on ALT-associated markers.

ALT activity is associated with elevated levels of telomeric recombination and repair, and the production of unusual telomeric structures. It is only starting to become clear which of these are associated directly with ALT telomere elongation, and which are required for rectifying ALT-associated telomeric DNA damage or are by-products of the changes required to promote the activation of ALT, and hence are less tightly linked to the ALT mechanism. ALT appears to be a homologous-recombination-directed DNA replication process [2] that requires PML-dependent localisation of the BLM-TOP3A-RMI (BTR) complex to telomeres [3]. This co-localisation usually occurs in PML nuclear bodies, which, with the presence of telomeric DNA, are called ALT-associated PML bodies (APBs), a useful single cell ALT marker [4,5,6]. Another characteristic of ALT is heterogeneous telomere lengths ranging from (telomere) signal-free chromosome ends to abnormally long telomeres [2,4,7,8]. Both telomeres that are too short to be functional and those that are too long to avoid replication fork stalling could be sources of the increased DNA damage signals seen in the ALT+ telomeres [9,10]. ALT is also associated with elevated telomeric recombination, which is largely due to the loss of the ALT suppressor, ATRX [11]. Another feature of ALT is elevated levels of extrachromosomal telomeric repeats (ECTR) and epigenetic silencing of STING1 to prevent the activation of innate cellular immunity through CGAS sensing of ECTR [12,13]. One type of ECTR associated with ALT activity is the C-Circle, a partially single stranded and circular molecule of C-rich telomeric DNA that is preferentially derived from lagging strand replicated telomeres [14,15].

The C-Circle is an ALT specific biomarker that is tightly linked with the ALT telomere elongation activity. C-Circles are elevated in all ALT+ cell lines, including ALT+ cell lines lacking APBs, long heterogeneous telomeres, and t-circles (another species of circular ECTR) [14], and have not been detected in any mortal or telomerase+ cell lines, even in one induced to have APBs, long heterogeneous telomeres and t-circles [14]. C-Circles also appear when ALT is activated and decay rapidly after the inhibition of ALT, which makes them useful for screening for changes in ALT activity [14,16]. C-Circles appear to be more tightly linked to ALT telomere elongation than the telomere hyperrecombination phenotype of ALT+ cells. Complete knockout of PML or BTR provided clear inhibition of the ALT activity (clonal telomeres gradually shortened indicating lack of telomere maintenance) and corresponded with a five-fold decrease in C-Circles, but the telomere hyperrecombination (including telomeric sister chromatid exchanges) persisted unchanged [3]. Loss of ATRX (and similarly loss of STAG2 [17]) does not activate ALT or produce C-Circles but does increase telomeric recombination (including increased T-SCE) and generates other ECTR species including G-circles [11,18]. Further evidence that C-Circles are tightly linked to ALT telomere synthesis is that the knockdown of POLD3 or aphidicolin treatment decreases both the ALT activity and C-Circles [19,20]. However, C-Circle levels in ALT+ cells may not always reflect the level of ALT activity, as DNA damage and replication stress can increase C-Circle levels without net telomere elongation [16,21]. Nevertheless, C-Circles are perhaps the most ALT-specific of the available ALT markers and may be useful clinically. They are measurable by a quantitative assay [14] and better define a clinically distinct ALT+ group of neuroblastomas than another ALT marker, long heterogeneous telomeres [22].

C-Circles have been detected in the blood of patients with ALT+ osteosarcoma [14], and if they are protected from blood nucleases by being inside extracellular vesicles (ECVs) like the telomeric RNA, TERRA [23], they could provide a robust blood-based biomarker for a clinical diagnostic. Although ALT+ cancer cells have silenced CGAS-STING1 and/or ATRX sensing of ECTR [12], the C-Circles accumulating in ALT cell cytoplasm are likely to require degradation or excretion from the cell. If C-Circles are sorted into cytoplasmic multivesicular bodies they could be sent to lysosomes and/or excreted in exosomes, the latter of which can be taken up by the surrounding normal cells (with functional CGAS-STING1 and ATRX), which may elicit a type I interferon response and cytokine secretion [12,13] that could support nearby tumor growth. ECVs are categorized into three groups: (i) exosomes, which are formed by intraluminal budding of multivesicular bodies (MVBs) with contents selected by the endosomal sorting complex required for transport (ESCRT), which are secreted into extracellular spaces through the fusion of MVBs and plasma membrane and comprise most of the small EVs (50–150 nm diameter); (ii) microvesicles (MVs), which are also budded from the plasma membrane and comprise most of the medium-sized EVs (100–1000 nm diameter); and (iii) apoptotic bodies, which are budded from plasma membrane during cell apoptosis and are mostly the largest ECVs (>1000 nm diameter) [24,25,26,27,28,29]. Studies have shown that cancer cell-derived EVs are capable of shuttling oncogenic proteins and genetic materials between cells, promoting the proliferation, invasiveness, chemoresistance, and stemness of cancer cells [30,31,32,33,34]. In this study, we provided the first evidence that C-Circles are secreted in the exosomes from the conditioned media of ALT+ cancer cells, and we demonstrate that the C-Circle assay (CCA) can provide an accurate blood-based diagnostic for ALT+ cancer. 

## 2. Materials and Methods

### 2.1. Cell Culture and Patient Specimens

Human cancer cell lines G-292, SaOS-2, SK-LU-1, U-2 OS, ZK-58, A549, HT1080, MG-63, SH-SY5Y, SK-N-BE2c, SJSA-1, TE-85, and U-251 and normal fibroblast HFF5 were grown in Dulbecco’s Eagle Medium (DMEM) supplemented with 10% fetal bovine serum (FBS) and 1× Glutamax (ThermoFisher, Scoresby, Australia). Human cancer cell lines DOS16, SK-N-FI, YTBO, and LA-N-6 were grown in Roswell Park Memorial Institute (RPMI) 1640 medium with 10% FBS and 1× Glutamax (ThermoFisher). Human cancer cell lines CHLA-90 (passage 382) and COG-N-291 (passage 347) were grown in Iscove’s Modified Dulbecco’s Media (IMDM; ThermoFisher) with 1× ITS (Insulin-Transferrin-Selenium; Sigma (Macquarie Park, Australia)). All of the cells were grown in a humidified incubator at 37 °C with 5% CO_2_, confirmed negative for Mycoplasma contamination and confirmed >90% viable cells (Trypan Blue exclusion; ThermoFisher) before each experiment; the cell origins and sources are provided in Table S1. Ethics approval for the study was obtained from the Sydney Children’s Hospital Network and Northern Hospital Network Human Research Ethics Committees. The study cohort consisted of 35 high-risk neuroblastoma cases from the Children’s Oncology Group Neuroblastoma Biobank. High-risk neuroblastoma was defined as metastatic disease in patients aged ≥12 months or as tumors with MYCN amplification irrespective of the disease stage. All of the samples were obtained at original diagnosis after informed consent at the institution where the patients were diagnosed was obtained.

### 2.2. Differential Centrifugation of Extracellular Vesicles (ECV)

**Figure 1:**
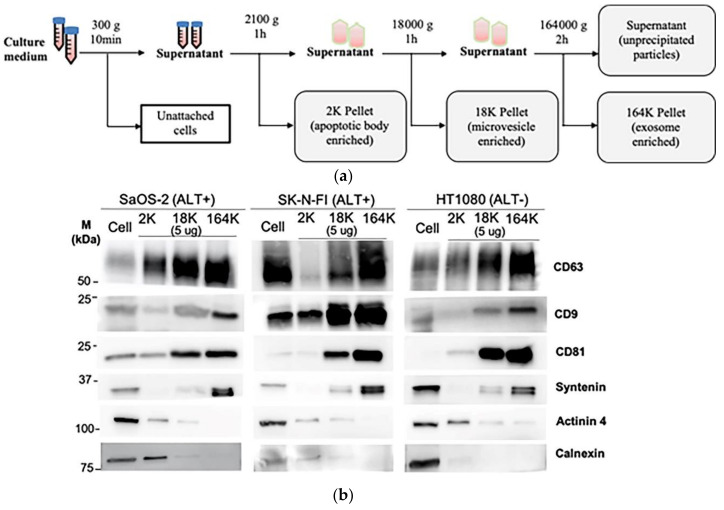

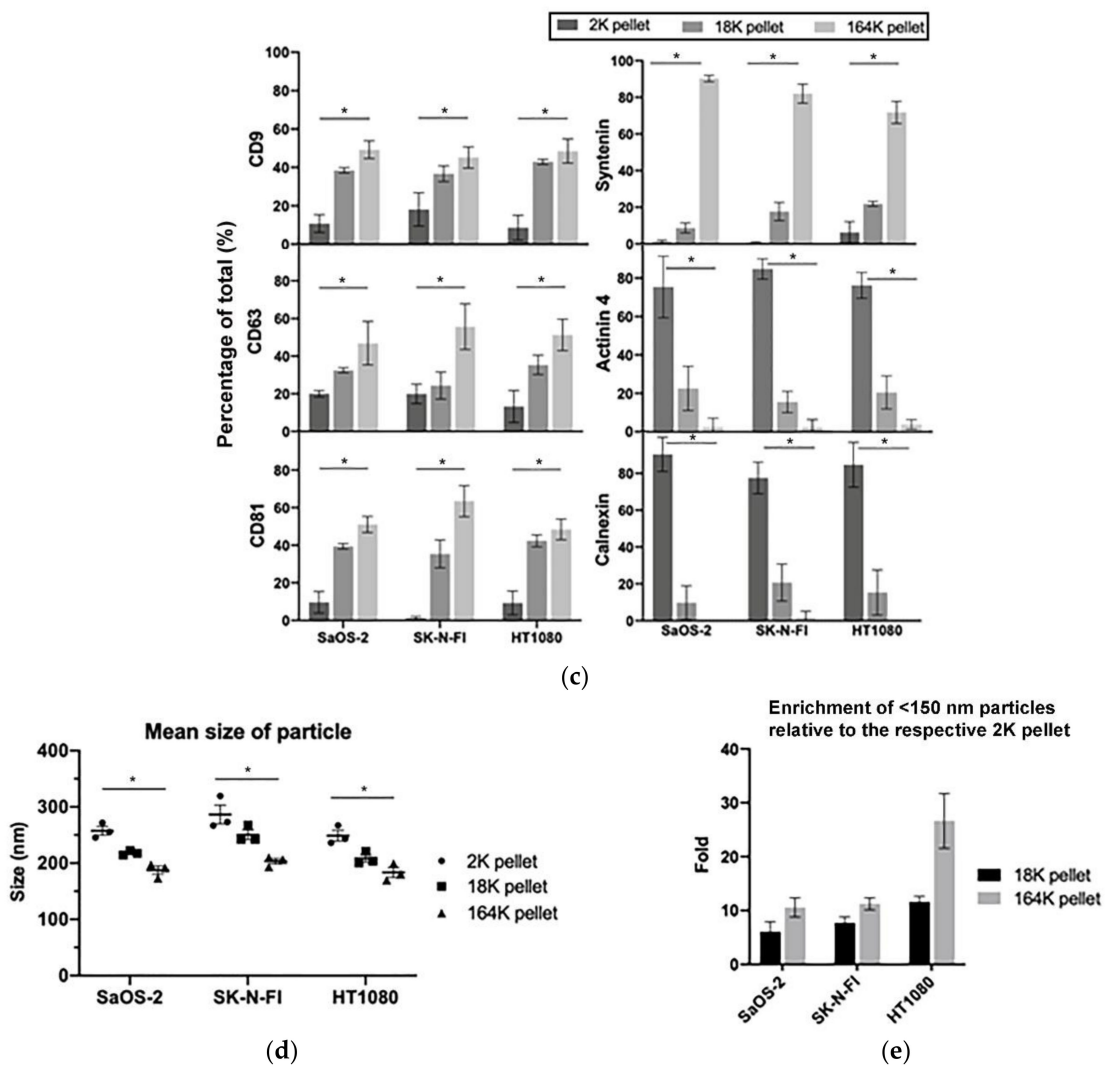
Isolation of extracellular vesicles (ECVs) by differential centrifugation: (**a**) schema of the differential centrifugation for EV collection. (**b**) A representative Western blot of the successive 2K, 18K, and 164K pellets obtained by differential centrifugation from SaOS-2, SK-N-FI, and HT1080 (5 μg loaded from each pellet), with the whole cell protein lysates (30 μg) of the corresponding cell lines were loaded as positive controls. (**c**) Quantitation (mean) of three Western blot replicates for each of CD9, CD63, CD81, Syntenin, Actinin 4, and Calnexin; error bars indicate the standard error of mean (SEM). (**d**) Nanoparticle tracking analysis determined mean ECV size in the 2K, 18K, and 164K pellets from SaOS-2, SK-N-FI, and HT1080 (*n* = 3, error bars show SEM). (**e**) Fold enrichment of exosomes in the 18K and 164K pellets relative to their respective 2K pellets (proportion of <150 nm particles in the pellet)/(proportion of <150 nm particles in the 2K pellet) for SaOS-2, SK-N-FI, and HT1080 (*n* = 3, error bars show SEM). * *p* = 0.014 by the Friedman test, Bonferroni corrected significance threshold was 0.017.

Secreted ECVs were collected over 24 h in fresh bovine-ECV-depleted culture media (ultracentrifuged at 100,000× *g* and 4 °C for 16 h) that had been placed on cells washed three times with phosphate buffered saline (PBS; ThermoFisher). ECVs were isolated from the conditioned (24 h old) media by differential centrifugation [35]. Conditioned media was spun at 300× *g* for 10 min, 2100× *g* for 60 min, 18,000× *g* (18K) for 60 min, and 164,000× *g* (164K) for 120 min, as described in Figure 1. All spins were performed at 4 °C and the last two were performed (18K and 164K) in a 70Ti rotor (Beckman, Cove West, Australia). Pellets were washed with PBS (resuspended and centrifuged at the same speed for 120 min) and stored resuspended in 50–100 μL of PBS at −80 °C. After harvesting their conditioned media, the cells were detached and pooled with the 300× *g* pellet to be assessed for cell viability with Trypan Blue staining on a TC10 Cell Counter (Bio-Rad, Gladesville, Australia). Less than 10% cell death was observed in the cells used for these studies.

### 2.3. Iodixanol Gradient Separation of Evs

First, 164K pellets collected by differential centrifugation from 8–15 × 10^7^ cells were resuspended in 1.1 mL of STE buffer (0.25 M sucrose, 10 mM Tris-HCl, 1 mM EDTA, pH 7.4) and transferred to the tube of SW60Ti rotor (Beckman) and mixed 1:1 with 60% (wt/vol) stock solution of iodixanol (Optiprep medium, Sigma). STE buffer diluted iodixanol solutions, 1 mL of 20% iodixanol, and 0.8 mL of 10% iodixanol were layered sequentially on the top and the tube centrifuged at 350,000× *g* and 4 °C for 1 h (brake off). Eight fractions of 480 μL were collected from the top of the tube. For Western blot and CCA in the downstream analyses, 320 μL and 160 μL (respectively) from each 480 μL fraction was diluted with 2 mL and 240 μL PBS (respectively) and centrifuged at 164,000× *g* for 30 min in TLA110 and TLA 120.1 rotors (Beckman; respectively). For Western blot and immuno-isolation in the downstream analyses, each fraction was split into two 240 μL portions, diluted with 2 mL PBS for Western blot and 560 μL PBS for immuno-isolation, followed by centrifugation in TLA110 and TLA 120.2 rotors (Beckman), respectively. The pellets were resuspended in PBS for immuno-isolation, radioimmunoprecipitation assay (RIPA) lysis buffer (Cell Signaling) for Western blot, or Quick C-Circle Preparation (QCP) lysis buffer [16] for CCA, and stored at −20 °C.

### 2.4. Immuno-Purification of ECV

Mouse monoclonal antibodies, CD63 (catalogue number 556019, BD Biosciences, North Ryde, Australia), CD9 (catalogue number CBL162, Millipore, Bayswater, Australia), CD81 (Clone M38, Invitrogen (Scoresby, Victoria, Australia)), or normal mouse polyclonal IgG (catalogue number 12-371, Millipore) were used to coat magnetic beads (Pierce Protein A/G Magnetic Beads; ThermoFisher) at a ratio of 5 μg antibody:100 μL beads, as described by the manufacturer. The 164K pellet (derived from differential ultracentrifugation of conditioned media from 10^7^ cells) resuspended in 25 μL PBS was added to antibody-coated beads resuspended in 475 μL of TBST, and the mixture rotated overnight at 4 °C. For immuno-isolation of the density gradient purified exosomes only, 25 μL of the selected fraction in PBS was added to the antibody-coated beads and rotated at 4 °C overnight. Bead-bound exosomes were collected and washed three times with 500 μL TBST. The unbound supernatant was transferred to new tubes of TLA 120.1 rotor (Beckman) and centrifuged at 164,000× *g* for 30 min to obtain concentrated flowthrough (FT). Bead-bound exosomes and concentrated FT were lysed in 25 μL of 1× LDS loading buffer (Life Technologies) and heated for 10 min at 98 °C for the Western blot. 

### 2.5. DNA Extraction/Preparation and Nuclease Protection Assay

Quick C-Circle Preparation (QCP) or QIAamp blood DNA mini kit (QIAGEN, Chadstone centre, Australia) were used for DNA preparation/extraction in this study. DNA extraction from cells or ECV by QCP lysis was performed as previously described [16]. Briefly, the cells or ECV pellets were lysed in a QCP lysis buffer with Protease Q at a ratio of 1:20 at 56 °C with shaking at 900 rpm for an hour, and Protease Q heat-inactivated at 70 °C for 20 min at 900 rpm. DNA was extracted from the solutions and serum by QIAamp columns as described by the manufacturer with the DNA eluted in 10 mM Tris (pH 7.6) and stored at −20 °C before use. For the nuclease protection assay only, 500 ng DNA (cells) or DNA extract that corresponded to the input conditioned media harvested from 2 × 10^7^ cells (164K pellets) was treated with 1 unit of nuclease (Dnase I, NEB), incubated at 37 °C for 10 min, followed by the addition of EDTA (final 5 mM, pH 8.0) and inactivation at 75 °C for 10 min, after which it was stored at −20 °C before use and 1/3 of each reaction loaded into the C-Circle assay.

### 2.6. C-Circle Assay

C-Circles were detected by the C-Circle assay (CCA), as previously described [16], using 10 ng of DNA prepared from the cells. For the ultracentrifugation, density gradient, and immuno-purification fractions, a volume of DNA preparation was loaded into the CCA, which corresponded to input conditioned media from 3 × 10^4^–4 × 10^5^, 4–5 × 10^6^, or 5 × 10^5^ cells, respectively. Equal volume amounts of QCP lysate were loaded for all samples and the final CCA level corrected for the corresponding number of cells. C-Circle signals were amplified at 30 °C by phi29 DNA polymerase (7.5 unit/sample; without dCTP) for 8 h, which was heat-inactivated for 20 min at 65 °C. The products were diluted with 40 μL of 2× SSC and loading on a nylon membrane (Biodyne B+, Pall using a dot blot manifold (Whatman Minifold I 96-well system) according to the manufacturer’s instructions. DNA was UV-cross-linked onto the membrane, which was then hybridized with end-labelled ^32^P-(CCCTAA)_3_ in a 15 mL hybridization buffer at 37 °C. Phosphor screen exposure was optimised for its linear range and was scanned on an optical imager (FLA7000, GE Healthcare Life Science). The intensity of the dot blots was quantified using ImageQuant software version 8, using edge subtraction for the background correction. For detection by qPCR, 1.7 µL of CCA product was used with the Rotor-Gene Q (Qiagen) real-time cycler [22] and results expressed as mean of triplicates. Telomere qPCR: 95 °C, 15 min; 30 × (95 °C, 7 s; 58 °C for 10 s). Single-copy gene (SCG; VAV2) qPCR: 95 °C; 5 min, 43 × (95 °C, 15 s; 61 °C, 30 s; 72 °C for 20 s). Using the two standard curves method, telomeric DNA was normalized (norm-TEL) with the SCG. Relative C-Circle level = (norm-TEL − (norm-TEL from the Φ29-omitted control)) normalized to the C-Circle level of SKN-FI and IIICF/c cell lines with arbitrary values of 196 and 100, respectively [22].

### 2.7. Nanoparticle Tracking Analysis

EV size and concentration were determined by NanoSight (NS300, Malvern Panalytical (Taren Point, Australia)) according to the manufacturer’s instructions. Resuspended differential centrifugation pellets were diluted to 1 mL with PBS. Polystyrene microspheres (0.1 μm; Malvern Panalytical) were used to standardize the camera setting in each experiment (camera level 11). At least four videos of 60 s were recorded for each sample dilution and were analyzed using NanoSight NS300 NTA software 3.2 (Malvern Panalytical).

### 2.8. Western Blotting

Equal micrograms of protein from the differential centrifugation pellet protein lysate (RIPA buffer) were mixed with non-reducing 4× LDS sample buffer (Thermo Fisher), heated at 98 °C for 10 min, and kept on ice briefly before loading on 10% Mini-protean TGX Precast gels (Bio-Rad) under non-reducing conditions. The proteins were transferred to Immobilon-P PVDF membranes (Millipore), which was then blocked with 5% non-fat milk in 1× TBST (1× TBS-0.1% Tween 20). Primary antibodies (1:1000) were incubated with membranes overnight at 4 °C, and secondary antibodies, anti-mouse, and anti-rabbit (1:2000, Cell Signaling) were incubated at room temperature for 1 h. Development was performed using SuperSignal^TM^ West Pico Plus Chemiluminescent Substrate (Thermo Fisher) and detected by ImageQuant LAS 4000 (GE Healthcare Life Science).

### 2.9. Data Analysis

All analyses were blinded with respect to the previous results and patient data. Distributions were compared by the two-tailed Friedman test and Bonferroni correction for multiple comparisons (unless otherwise specified) with IBM SPSS Statistics software. All of the experiments were performed in triplicate and plotted as mean ± SEM, unless stated otherwise.

## 3. Results

### 3.1. Isolation of Exosomes and Microvesicles by Differential Centrifugation

To determine how C-Circles are secreted by cancer cells, we collected 24 h-old cell culture medium and used differential centrifugation to isolate the secreted extracellular vesicles (ECV), which include apoptotic bodies (AB), microvesicles (MV), and exosomes (EXO) [24,36]. Centrifugal force was increased stepwise to avoid fragmenting large vesicles, and AB, MV, and EXO were enriched in the 2100× *g* (2K) 18,000× *g* (18K), and 164,000× *g* (164K) pellets, respectively (Figure 1a). This was confirmed by Western blot analysis (Figure 1b,c) and nanoparticle tracking analysis (NTA; Figure 1d,e) for the ALT+ cell lines SaOS-2 and SK-N-FI and the telomerase+ cell line HT1080. As shown in Figure 1b,c, the exosome-specific proteins Syntenin, CD9, CD63, and CD81 [27,36,37,38] were concentrated in the 164K pellet, which indicates successful enrichment of the exosomes in the 164K pellet. Of the total amount of Syntenin found in the ECVs, an average of 90%, 9%, and 1% were found in the 164K, 18K, and 2K ALT+ pellets, respectively (and similarly for the telomerase+ HT1080 control; Figure 1b,c, Figure S1 and Table 1). Of the total amount of Tetraspanins (CD9, CD63, and CD81 combined), an average of 52%, 35%, and 13% were found in the 164K, 18K, and 2K ALT+ pellets, respectively (Figure 1b,c). Although CD9, CD63, and CD81 are standardly used as exosome markers, a substantial proportion are usually found in the 18K (MV-enriched) pellet and the average proportion, 35%, found in the 18K pellet is consistent with the results reported by other research groups [36]. Alpha-Actinin-4 (Actinin-4) is a plasma membrane associated protein and, on average, only 2% of the total Actinin-4 was in the ALT+ 164K pellets, which is consistent with there being minimal MV and AB in the 164K pellet [36] (Figure 1b,c; MV and AB are derived from the plasma membrane, unlike EXO). The endoplasmic reticulum protein, Calnexin, was absent (1%) in the ALT+ 164K pellets and only 15%, on average, of the total Calnexin was in the 18K ALT+ pellets, which is consistent with there being minimal AB in the 18K and 164K ALT+ pellets [36] (Figure 1b,c). NTA showed a progressive decrease in the mean particle size in the pellets as the centrifugation speed increased (Figure 1d), and that ECV with diameters <150 nm, which correspond to the size of the EXO [39], were more concentrated in all the 164K pellets than their corresponding 2K or 18K pellets (*p*-values 0.014 or 0.22, respectively; Figure 1d,e) and were enriched 10- to 30-fold in the 164K pellets relative to the 2K pellets (Figure 1e). The NTA size profiles of the ECV in the 164K pellets are shown in Figure S2. Our results, summarised in Table 1, demonstrate a similar level of enrichment of EXO and MV in the 164K and 18K pellets, respectively, to that reported in the literature [36,40].

### 3.2. C-Circles Are Secreted inside Exosomes in ALT+ Cancer Cell Lines

**Figure 2:**
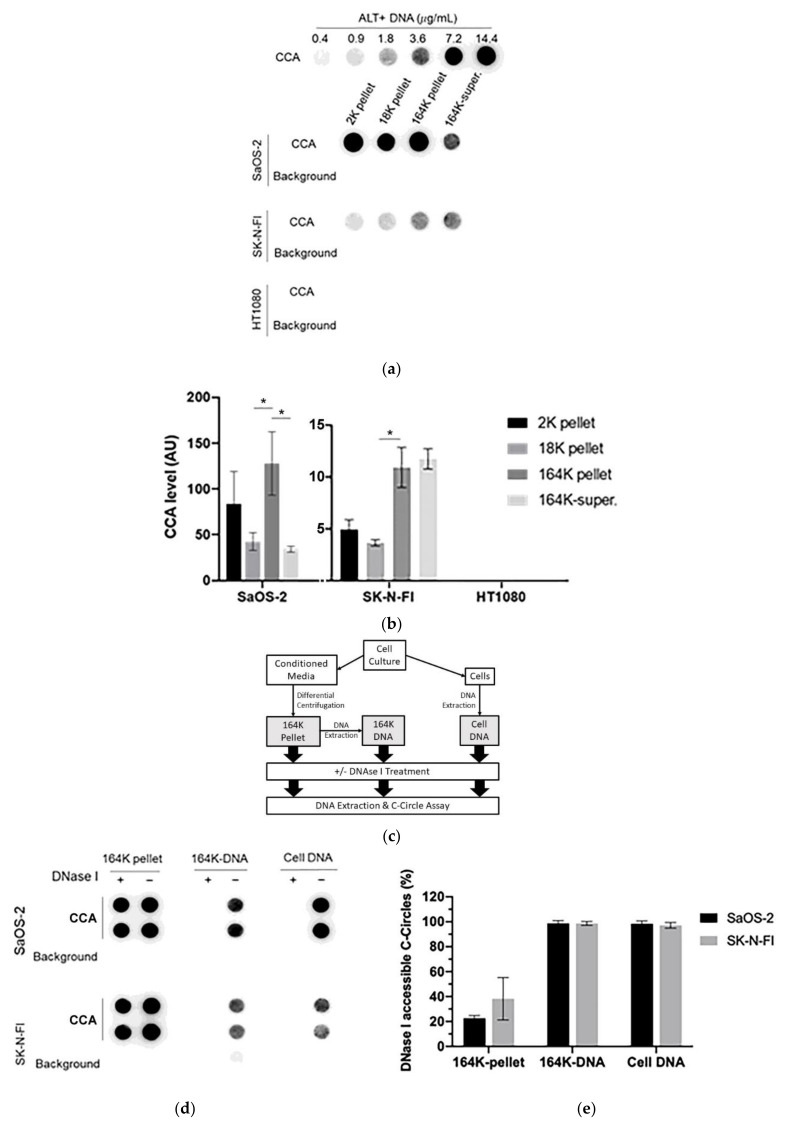
C-Circles are secreted inside the exosomes in the ALT+ cancer cell lines: (**a**) the three centrifugation pellets (2K, 18K, and 164K) and the 164K supernatant (164K super.) were analyzed with the C-Circle Assay (CCA; *n* = 3), and a representative dot blot is shown. A standard curve using ALT+ DNA (U-2 OS genomic DNA) was included to calibrate replicate blots and confirm linearity. The sample-specific background (Background) is the CCA performed omitting Phi29 DNA polymerase and measures the amount of pre-existing CCA product (single-stranded telomeric G-strand) [16]. (**b**) Quantitation of the CCA dot blots shows the extracellular C-Circle levels of SaOS-2 and SK-N-FI (*n* = 3; three independent experiments each with two CCA replicates) in the different centrifugation fractions. The scale on the vertical axis is different for SaOS-2 than for SK-N-FI and HT1080, * *p* < 0.013 by the Friedman test, Bonferroni corrected significance threshold was 0.017 (comparisons of the 164K-pellet to the other fractions only). (**c**) Schema of the DNase I protection assay performed on the 164K (EXO-enriched) pellet. The 164K (EXO-enriched) pellet of SaOS-2 or SK-N-FI were treated with Dnase I either before (164K pellet) or after (164K-DNA) the extraction of DNA for the CCA. The whole cell DNA extracts (Cell DNA) were used as the positive controls and sample-specific background (Background) checked by omitting the Phi29 DNA polymerase from the CCA. A representative dot blot is shown (**d**), and the quantitation of the replicates (*n* = 3) is plotted in (**e**); error bars indicate SEM. Overexposed CCA dot blots are presented in Figure S3 for (**a**,**d**) to allow for better visualization of the Background signal.

To determine if C-Circles were secreted by cancer cells inside the ECV, we performed the CCA on each differential centrifugation fraction of 24 h-old culture medium from the ALT+ cancer cell lines SaOS-2 and SK-N-FI and the ALT- cancer cell line HT1080, and only saw the CCA signal in the fractions from the ALT+ cells lines (Figure 2a,b). We found CCA signal in the ALT+ 2K pellets, which was expected because AB are known to contain genomic DNA. Compared with their respective 2K pellets, the CCA signal was reduced in the ALT+ 18K (MV-enriched) pellets and elevated in the 164K (EXO-enriched) pellets (*p* = 0.038 and 0.004 for SaOS-2 and SK-N-FI, respectively). The CCA signal was three-fold higher in the 164K pellet than the 18K pellet for both SaOS-2 (128 AU, 95% CI = ±84.5 AU compared to 42.7 AU, 95% CI = ±22.9 AU) and SK-N-FI (10.9 AU, 95% CI = ±4.7 AU compared with 3.65 AU, 95% CI = ±0.7 AU; Figure 2b). As summarised in Table 1, these results are consistent with ALT+ cancer cells secreting C-Circles inside the exosomes. The 19% of CCA signal that was in the ALT+ MV-enriched 18K pellet (average) could be completely accounted for by the 9% of EXOs (ALT+ average Syntenin component) and 15% of Abs (ALT+ average Calnexin component) remaining in the MV-enriched 18K pellet.

To confirm that the exosome-associated C-Circles were inside exosomes and not adhering to the outside of exosomes, we pre-treated the 164K (EXO-enriched) pellets with Dnase I (Figure 2c). Dnase I completely digested the C-Circles that had been extracted from the exosomes or cells, but when incubated with the resuspended 164K pellet from SaOS-2 and SK-N-FI, Dnase I treatment only reduced the CCA signal by 23% (95% CI = ±2.1) and 38% (95% CI = ±17.0), respectively (Figure 2d,e). This is consistent with the majority (60–75%) of 164K pellet C-Circles being protected inside the exosomes. Dnase I digestion of some of the C-Circles in the resuspended 164K pellets could have been due to the compromised integrity of the exosomes and the leakage of contents, or from a minority of extracellular C-Circles not being vesicles.

### 3.3. A Diverse Panel of ALT+ Cell Lines Secrete C-Circles in Exosomes

**Figure 3:**
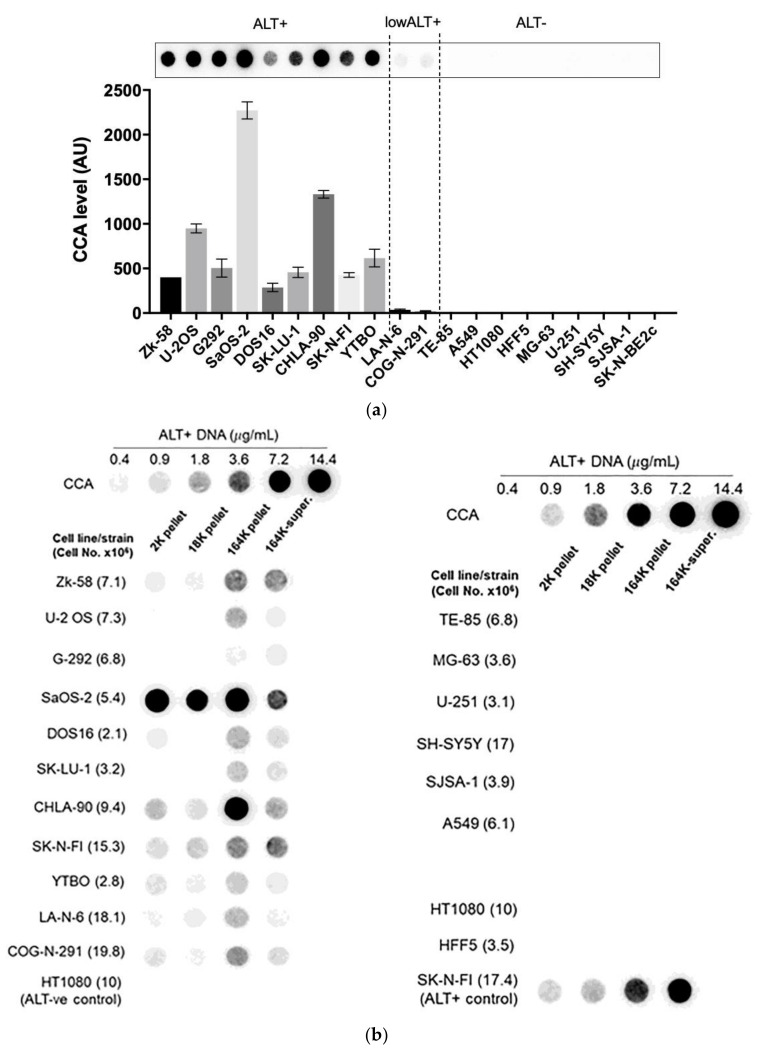

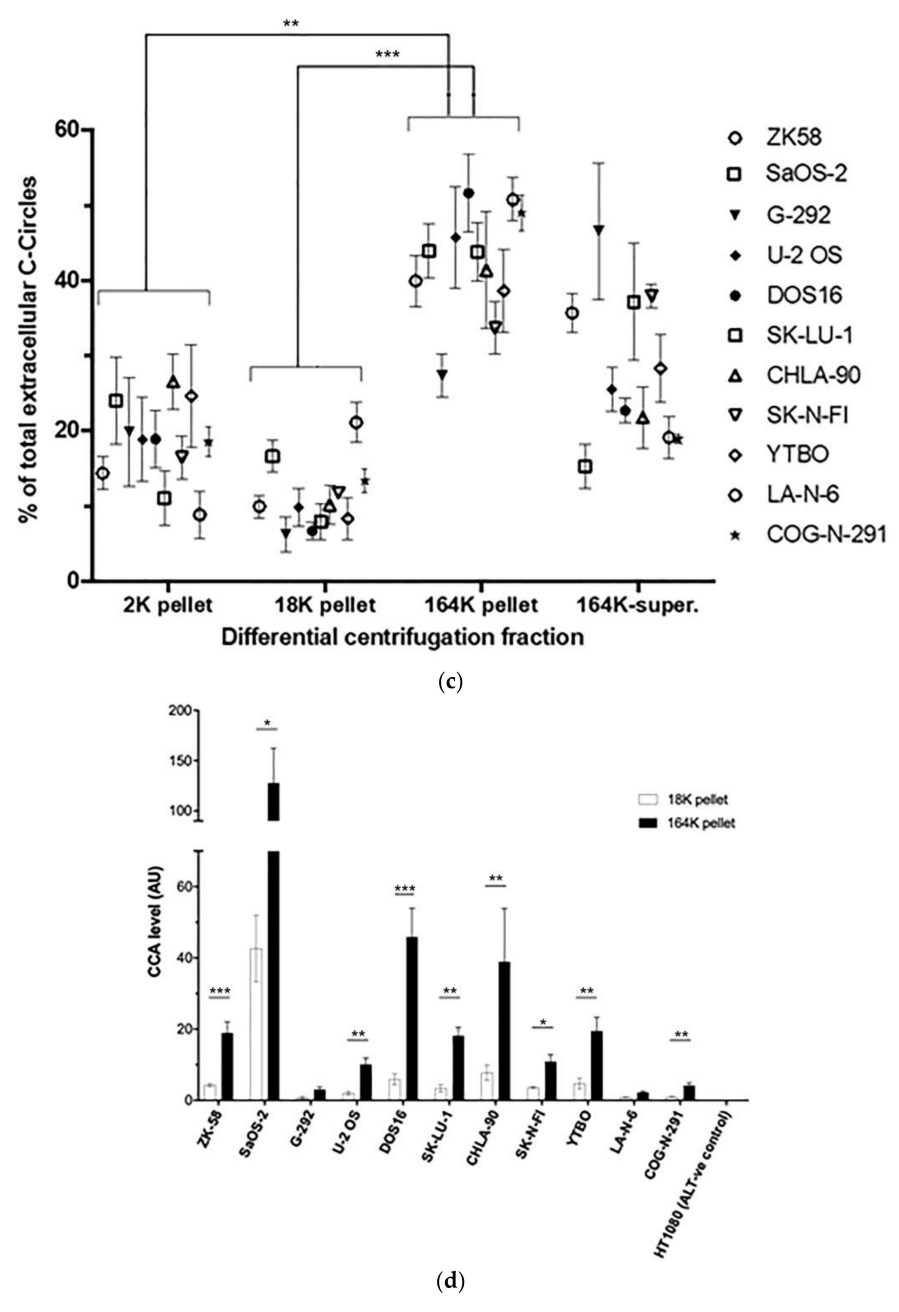

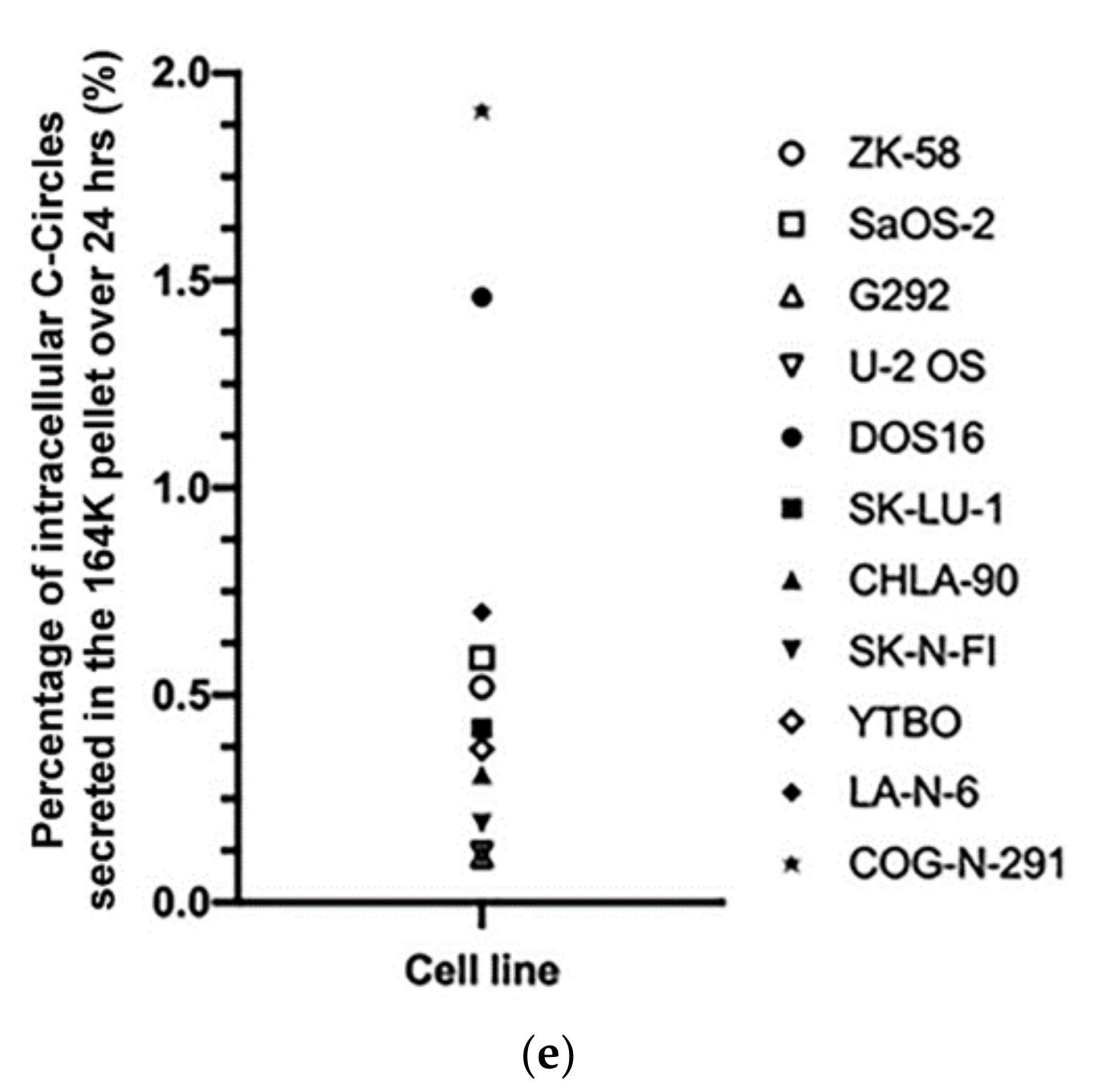
A diverse panel of ALT+ cell lines secrete C-Circles in exosomes: (**a**) CCA on whole cell DNA from a panel of ALT+ and matched ALT- cancer cell lines and strain, with a representative dot blot above the column graph (*n* = 3; Arbitrary Units, AU). All of the ALT+ cell lines had appreciable C-Circle levels, whereas no signal was observed in the ALT- cells. LA-N-6 and COG-N-291 are unusual ALT+ cell lines known to have low levels of ALT markers, including C-Circles [22]. (**b**–**e**) C-Circle levels in the differential centrifugation fractions of 24 h old culture medium from three independent experiments (duplicate CCA for each experiment) for each cell line/strain. (**b**) Representative CCA dot blots for the differential centrifugation fractions of each cell line. The cell numbers counted in the flask after culture media collection (indicated in parentheses) are used to normalize the CCA signals. A standard curve using ALT+ DNA (U-2 OS genomic DNA) is included at the top of each blot. (**c**) The CCA level in each differential centrifugation fraction for each ALT+ cancer cell line is presented as a percentage of the total (combined) CCA signal from all extracellular fractions (*n* = 3, error bars indicate SEM). (**d**) The absolute CCA values (arbitrary units (AU)) are graphed for the 18K and 164K pellets only (*n* = 3, error bars indicate SEM). (**e**) Plot of the percentage of the intracellular C-Circles that were secreted in the 164K (EXO-enriched) pellet for 24 h for each ALT+ cell line (*n* = 3 for both the 164K pellets and whole cell DNA; (mean of extracellular C-Circles in 164K pellet)/(mean of intracellular C-Circles)). * *p* < 0.013, ** *p* < 0.002, and *** *p* < 0.0003 by the Friedman test, Bonferroni corrected significance thresholds were 0.017 (comparisons of the 164K-pellet to the other fractions only).

We confirmed that these results extended to ALT+ cancer cell lines in general by performing the CCA on the differential centrifugation fractions of 24 h-old culture medium from a diverse panel of 11 ALT+ cancer cell lines and nine matching ALT− cells, including osteosarcoma, soft tissue sarcoma, neuroblastoma, glioblastoma, and non-small cell lung cancer cell lines, and a mortal cell strain. Included in this panel were two unusual ALT+ neuroblastoma cell lines, LA-N-6 and COG-N-291, which had only low levels of ALT markers including C-Circles [22]. These two cell lines had just converted to fully functional ALT activity after an extended time in the culture of apparent episodic ALT activity. The intracellular C-Circle levels for all cell lines and strains are shown in Figure 3a, and the extracellular fraction C-Circle levels are shown in Figure 3b–d and Figure S4 and Table S2. No ALT-negative cell line or strain showed a significant CCA signal in the extracellular fractions, which demonstrated that secreted C-Circles, like intracellular C-Circles, are specific for ALT. All ALT+ cancer cell lines had elevated CCA levels in the 164K (EXO-enriched) pellet (overall significantly higher than the 2K pellet, *p* = 0.002; Figure 3c). The ALT+ CCA signal in the 164 K pellet on average comprised 43.1% (95% CI = ±2.3%) of the total extracellular CCA signal, which was four-fold the average CCA signal in the 18K pellet (10.9% of total extracellular signal, 95% CI = ±1.2%; Figure 3c). Of the total CCA signal that was demonstrated to be secreted by ALT+ cancer cells in vesicles (MV or EXO), on average 79.8% (95% CI = ±3.5%) was found in the 164K (EXO-enriched) pellet.

A substantial proportion of the extracellular CCA signal continued through to the supernatant of the 164K centrifugation (26.3% on average, 95% CI = ±9.6%; Figure 3b,c), which could be due to free C-Circles and/or C-Circle containing exosomes that had not precipitated in the 2 h 164K spin. No difference in the overall exosome size profile was noticed between SaOS-2 and SK-N-FI (Figure S2), which had low and high relative CCA levels in the 164K supernatant, respectively.

After 24 h collection of the culture media, the ALT+ cells had secreted an average of 0.37% of their total intracellular CCA signal (95% CI = ±0.21%; *n* = 9; Figure 3e) into the EXO-enriched 164K pellet. This analysis did not include DOS16 or COG-N-291, which were outliers that secreted four- to five-fold more of their intracellular C-Circles (1.5% and 1.9%, respectively; Figure 3e). This may reflect the differences between the ALT+ cell lines in the mechanism or regulation of C-Circle secretion inside the exosomes. SaOS-2 showed the strongest secreted C-Circle signals per cell (Figure 3d), and we therefore used SaOS-2 for the downstream analyses. 

### 3.4. Density Gradient Purification Confirms C-Circles Are Secreted in Exosomes

**Figure 4:**
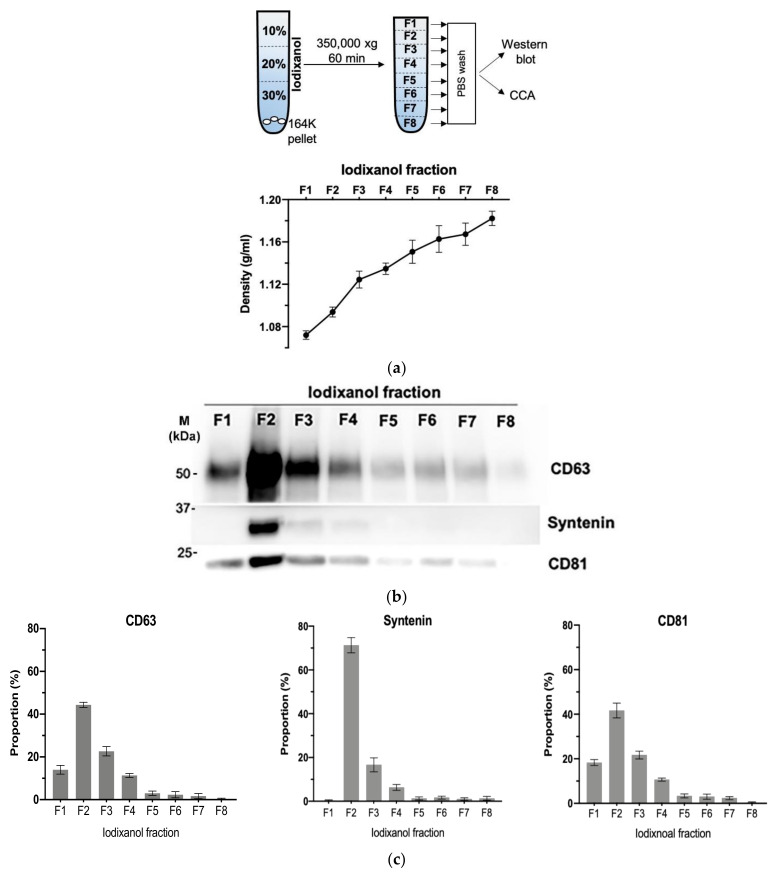

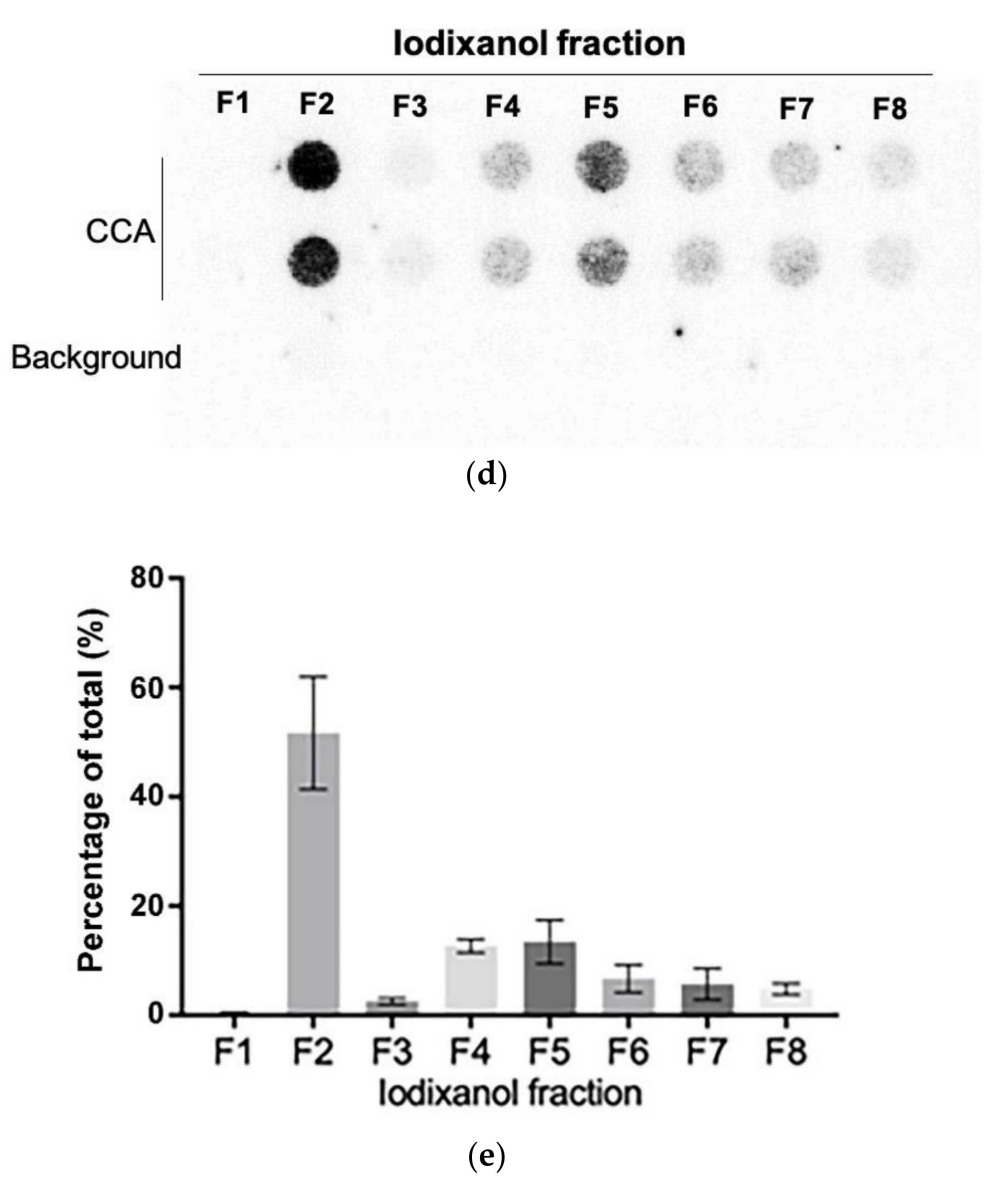
Purification of the exosomes by iodixanol density gradient separation: (**a**) schema of the iodixanol density gradient separation method (upper panel) and the density distribution in each fraction collected (lower panel). (**b**) Representative Western blot of exosome-specific proteins *CD63*, *CD81*, and *Syntenin* in the iodixanol fractions. The positions of the protein size standards (*M*) are shown. (**c**) Quantitation (*n* = 3) of the Western blots presented as the percentage of the total combined signal for each fraction. (**d**) Representative C-Circle assay (CCA) dot blot of the iodixanol fractions, with the sample-specific background (Background) checked by omitting the Phi29 DNA polymerase from the CCA. An overexposed CCA dot blot is presented in Figure S3 to allow for better visualization of the Background signal. (**e**) Quantitation (*n* = 3) of the CCA presented as the percentage of the total (combined) CCA signal from all fractions. The major CCA peak was in F2, which contained 52% (95% CI = 10%) of the total CCA signal from all fractions. All error bars indicate the SEM.

We further purified the exosomes in the SaOS-2 164K pellet through iodixanol density gradient separation. This additional density-based purification better resolves exosomes from other vesicles and protein aggregates than sedimentation-rate-based purification alone [36]. Eight fractions were collected from the top of the tube for Western blot analysis and CCA (Figure 4a). The exosomal marker proteins, CD63, CD81, and Syntenin, had a well-defined single peak in the second fraction (Figure 4b,c and Figure S5; F2) with an average iodixanol density of 1.094 g/mL (Figure 4a), which is the same density as the exosome containing fraction reported in other studies [39]. The CCA signal also had a well-defined peak in F2 (Figure 4d,e), which confirms that the majority of C-Circles were enriched in the fractions containing exosomes. There was a second, minor and broader CCA peak (20% of the height of the major peak) spanning the fourth and fifth fractions (Figure 4e; F4 and F5). There was no detectable corresponding peak in CD63, CD81, or Syntenin, and negligible signal from these exosome-specific proteins in F5.

### 3.5. Immuno-Purification Confirms That Some C-Circles Are Secreted in Exosomes

**Figure 5:**
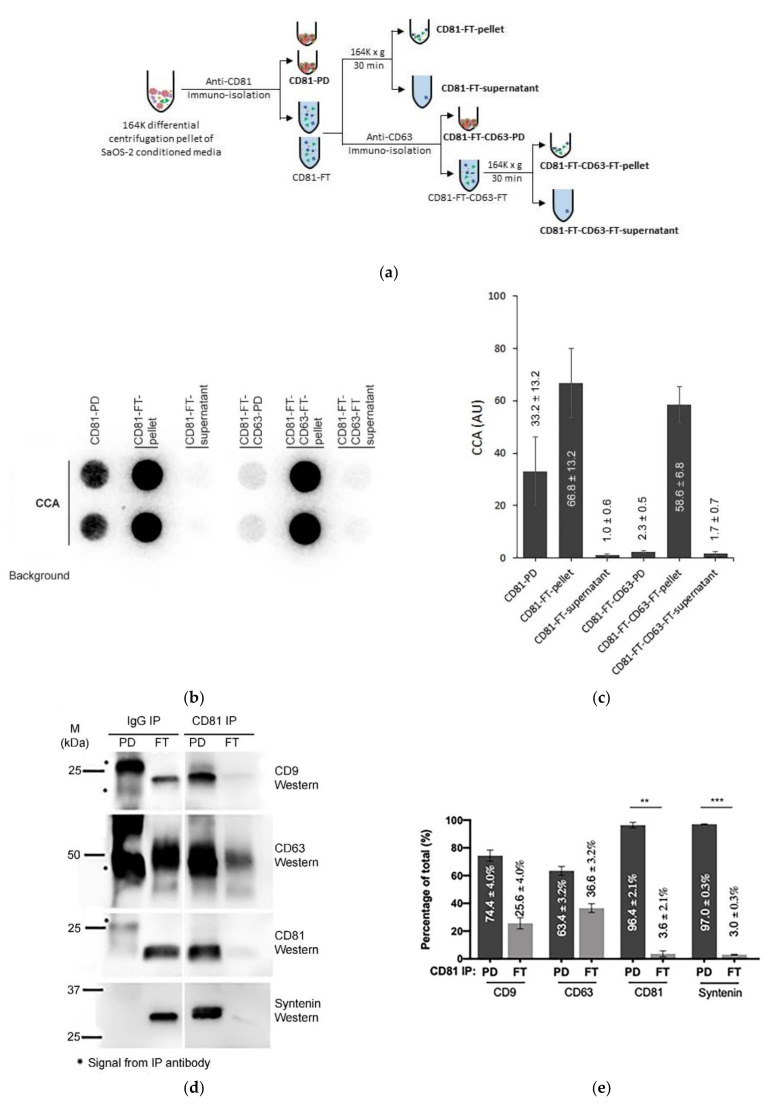

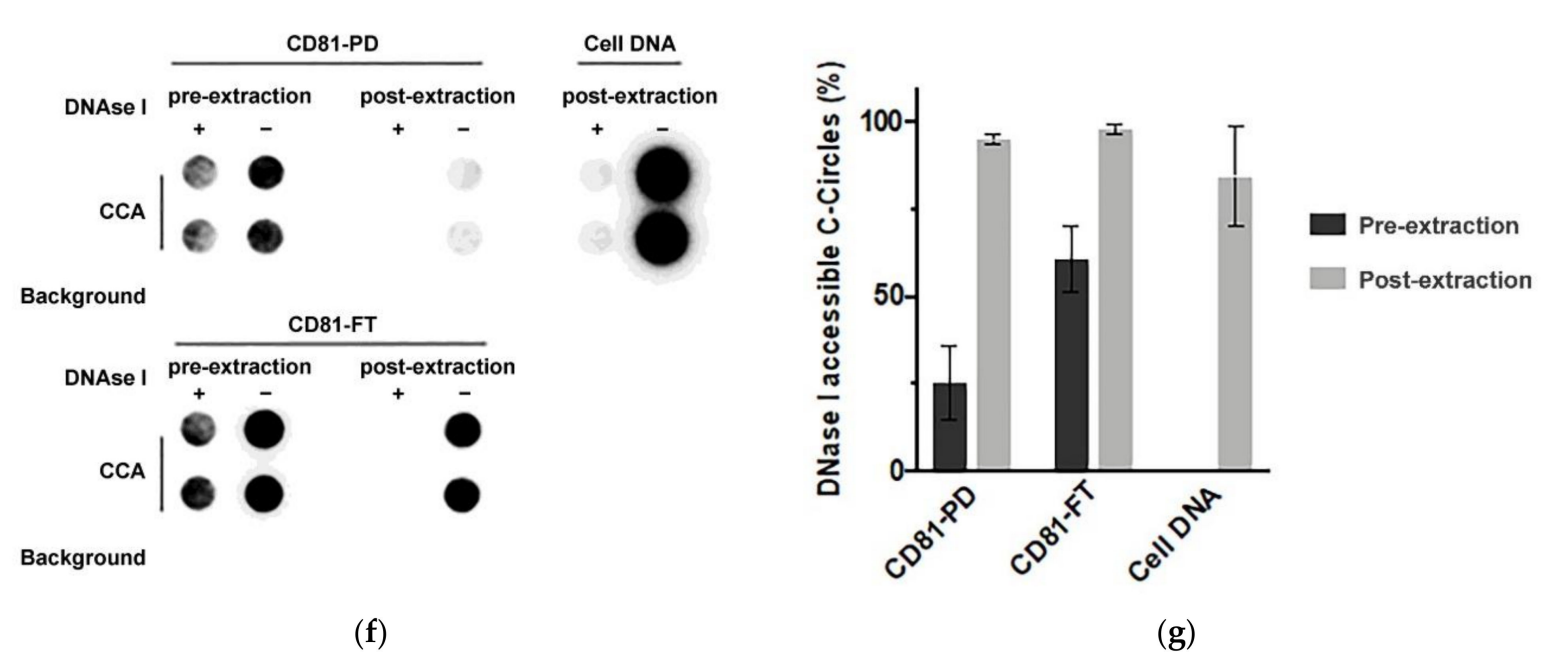
Immuno-purification of C-Circles with exosomes: (**a**) schema of the CD81 immunopurification method, fractions with names in bold font are analyzed by the C-Circle assay (*CCA*). (**b**) Representative CCA dot blot of the immunopurification fractions, with the sample-specific background (Background) checked by omitting the Phi29 DNA polymerase from the CCA. (**c**) Quantitation (*n* = 3) of the CCA presented as the percentage of the total CCA signal (CD81-PD and CD81-FT combined). (**d**) Representative Western blot of exosome-specific proteins *CD9, CD63*, *CD81*, and Syntenin in the CD81-PD and CD81-FT fractions. The positions of the protein size standards are shown. (**e**) Quantitation (*n* = 3) of the Western blots presented as the percentage of the total combined signal. CD81-PD or CD81-FT are treated with Dnase I either before (pre-extraction) or after (post-extraction) the extraction of DNA for the CCA. A representative dot blot is shown (**f**), with whole cell DNA (Cell-DNA) and sample-specific background (Background) controls. The quantitation of the replicates (*n* = 2) is plotted in (**g**), which shows that before DNA extraction, 25% (SEM = 9%) and 60% (SEM = 8%) of the CCA signal in the CD81-PD and CD81-FT, respectively, are accessible to DNAse I. All error bars indicate the SEM. Overexposed CCA dot blots are presented in Figure S3 for (**b**,**f**) to allow for better visualization of the *Background* signal. ** *p* < 0.002 and *** *p* < 0.0003 by the Friedman test.

We have demonstrated that extracellular C-Circles co-purify with exosomes by both differential and iodixanol gradient centrifugation, and therefore are part of entities that have indistinguishable sedimentation rates and densities to those of exosomes. These C-Circles are also protected from Dnase I digestion, consistent with being inside exosomes. To verify that C-Circles are secreted in exosomes, using a third method, we isolated exosomes from SaOS-2 164K (EXO-enriched) pellets by CD81 immuno-purification (Figure 5a; CD9 and CD63 immuno-purification produced similar results, data not shown). Half of the flow-through (FT) was separated by 164,000× *g* centrifugation into a pellet and supernatant, and the other half was immuno-purified with anti-CD63 and the resultant FT (CD81-FT-CD63-FT; Figure 5a) also separated into a 164,000× *g* centrifugation pellet and supernatant. All of the fractions were analyzed by the CCA (Figure 5b,c); the anti-CD81 pull down (PD) and FT were also analyzed by Western blot (Figure 5d,e and Figure S6).

Nearly all (97.0%; SEM = 0.3%) of the exosome-specific protein, Syntenin, was pulled down by the anti-CD81 antibody, and no detectable Syntenin signal was seen in the anti-IgG PD (Figure 5d,e), which indicates effective immuno-purification of the exosomes by the anti-CD81 antibody. Anti-CD81 pulled down 33.2% (SEM = 13.2%) of the CCA signal Figure 5c,d). This is similar to the 18–43% of the exosomal TERRA signal that is reported to be pulled down by anti-CD81 and anti-CD63 antibodies [23]. The CCA signal in the CD81 FT was not associated with the 36.6% (SEM = 3.2%; Figure 5d,e) of CD63 that escaped CD81 PD, and only 2.3% (SEM = 0.5%) of the CCA signal was in CD81-FT-CD63-PD (Figure 5b,c). The CCA signal in the CD81-FT could have resulted from degradation of the exosomes during the immuno-purification procedure, considering that there were negligible apoptotic bodies and microvesicles in the SaOS-2 164K pellet (Table 1) and therefore the CD81-FT, and that there was negligible Syntenin in the CD81-FT, which is tightly bound to exosomal membrane proteins (including tetraspanins) [41,42]. Performing the CD81 immuno-purification on the F2 fraction from Figure 4 did not increase the proportion of CCA signal in the CD81-PD (data not shown), and there are no known particles that have the same sedimentation rate and iodixanol buoyancy as exosomes but do not contain either Calnexin, Anexin-4, or Syntenin. Loss of exosome integrity during immuno-purification was supported by the Dnase I protection assay that found 2.4-fold more C-Circles accessible to Dnase I in the CD81-FT than in the CD81-PD or the 164K pellet (Figure 5f,g). The result that CD81 immuno-purification pulled down a similar proportion of C-Circles as reported for exosomal TERRA [23] confirms that at least some C-Circles are secreted in the exosomes; however, it remains to be determined if there is any association between exosomal C-Circles and exosomal TERRA.

### 3.6. Serum-Based CCA as an ALT Diagnostic in Neuroblastoma

**Figure 6:**
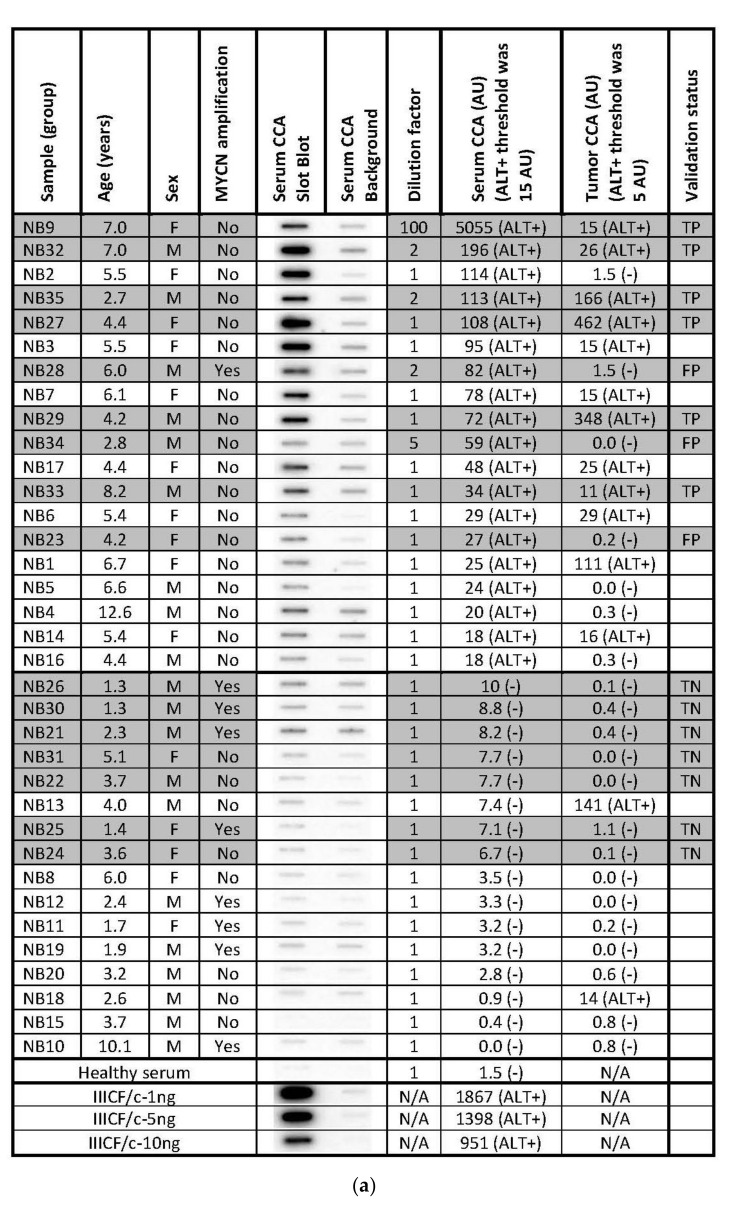

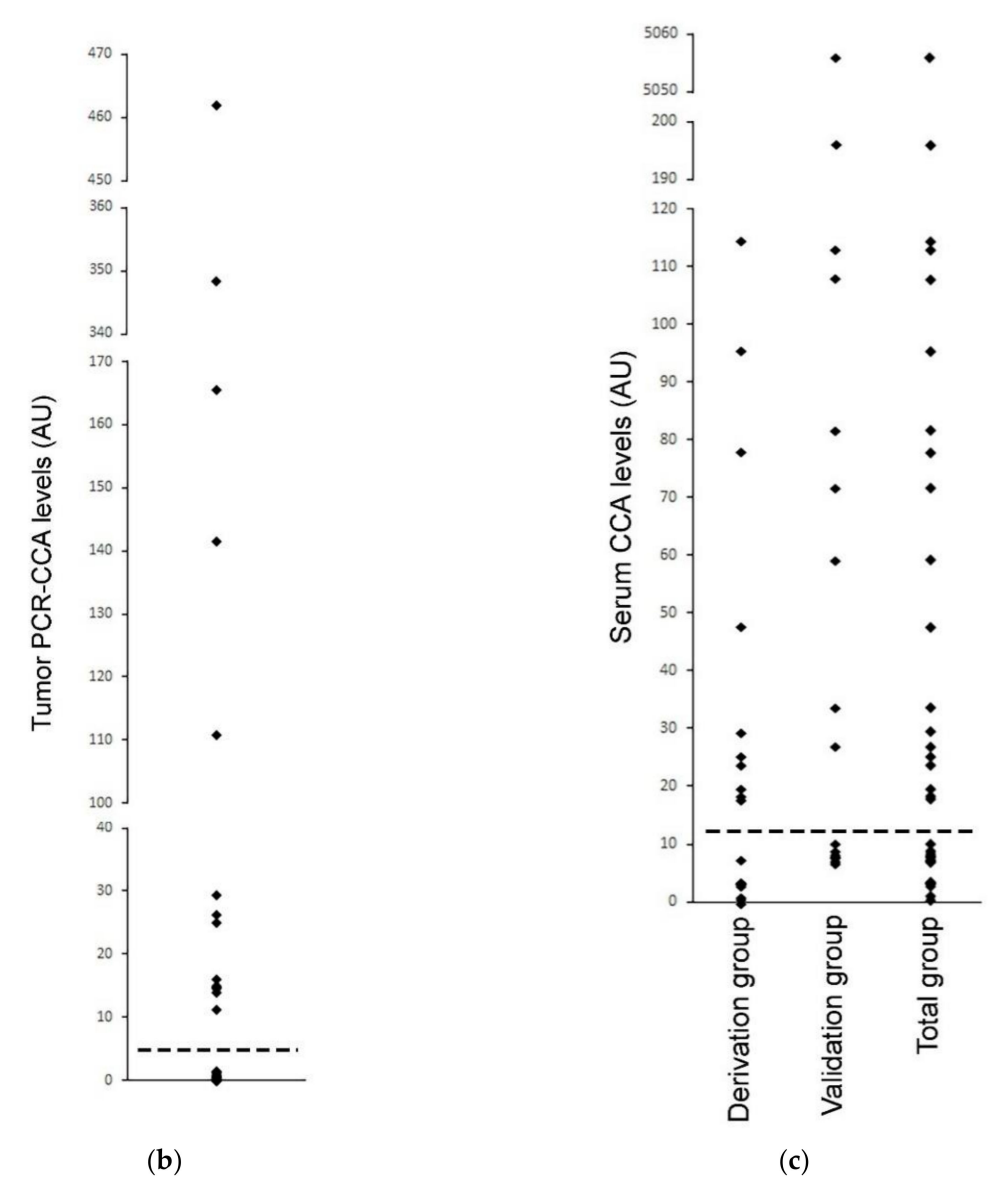
Serum-based CCA in the neuroblastoma. The CCA is performed on serum specimens from 35 cases of high-risk neuroblastoma, whose corresponding tumor specimens had already been tested for ALT by the PCR-CCA (alternate version of the CCA that uses qPCR for detection of the CCA products) [22]. (**a**) The Serum CCA Slot Blot results and the Serum CCA Background results (negative control with Phi29 DNA polymerase omitted) for the neuroblastoma serum specimens NB1-NB35 and a healthy control, and the ALT+ DNA controls from cell line IIICF/c. The same volume is loaded into the CCA for all of the serum sample DNA extracts, except for four cases that are diluted, and these are corrected for with the Dilution factor, as shown. The serum CCA result shown is the (CCA − background) × (dilution factor × interblot calibration); the interblot calibration factor is 1.1 for the shaded samples, which are on the (second) validation group CCA blot. The ALT+ threshold for the serum samples is determined at 12.2 arbitrary units (AU) by the (first) Derivation group CCA blot. The PCR-CCA results from the corresponding tumor specimens [22] are shown and their ALT+ threshold is 5 AU. True-positives (TP), false-positives (FP), and true-negatives (TN) in the Serum CCA Validation group with respect to the tumor PCR-CCA results are shown in the Validation status column. Patient age and sex, and tumor MYCN amplification status for each case are shown, as reported by Dagg et al. [22]. (**b**) Distribution of the PCR-CCA results from the 35 neuroblastoma tumor specimens [22], with the ALT+ threshold shown as a dotted line. (**c**) Distribution of the CCA results from the 19 neuroblastoma serum specimens in the Derivation group, 16 neuroblastoma serum specimens in the Validation group, and 35 neuroblastoma serum specimens in the Total group are plotted with the ALT+ threshold shown as a dotted line, whose level was determined from the Derivation group.

The secretion of C-Circles by cancer cells inside the exosomes could protect the secreted C-Circles from degradation by serum nucleases and allow them to be used as a blood-based biomarker for ALT+ cancer. To test if a blood-based CCA could be used as a diagnostic for ALT+ cancer, we performed the CCA on the serum specimens from 35 cases of high-risk neuroblastoma, whose matching tumor specimens had already been tested for ALT [22] by a version of the CCA that uses quantitative PCR for the detection of the CCA products (PCR-CCA) instead of dot blot [43]. PCR-CCA determined that 40% of the 35 high-risk neuroblastoma tumor specimens were ALT+ [22] and the individual results are shown in Figure 6a, with their distribution in Figure 6b. This group of 35 neuroblastoma cases that had matching serum available came from a larger group of 149 high-risk neuroblastoma tumor specimens, of which 24% were ALT+ [22]. The amount of DNA extracted from the serum specimens varied from 35 ng–1.5 mg per 400 μL, with no significant difference between the cases with ALT+ and ALT− tumors, with median extracted DNA concentrations of 166 ng/400 μL and 190 ng/400 μL for the ALT+ and ALT− groups, respectively (*p*-value = 0.7, student *t*-test). The same volume of the DNA extract was loaded into the CCA for all of the serum samples, except four samples where less was used and their CCA results were adjusted by a dilution factor (Figure 6a). It is not appropriate to adjust the serum samples for the DNA content, of which there is a variable contribution from the cancer cells and non-cancer cells. There was only enough sample to perform the CCA once with a background control (negative control) with Phi29 DNA Polymerase omitted to show the pre-existing CCA product [16], and the dot blot and the quantitation are shown in Figure 6a. 

The neuroblastoma cases were randomly assigned to either the derivation or validation groups, with the CCA for each group performed on separate dot blots calibrated by their standard curves (Figure 6a). The distribution of the serum CCA results in the derivation group (Figure 6c) showed two distinct subgroups, one subgroup with CCA levels less than 12.2 AU and the other subgroup with CCA levels above 12.2 AU. Therefore, 12.2 AU, which was eight-fold the CCA level of the healthy control serum (Figure 6a), was used as the threshold for diagnosing the serum specimens in the validation group as ALT+. Using this threshold, 9/16 (56%) of the neuroblastoma serum specimens in the validation group were ALT+ and the serum-based CCA had a sensitivity of 100% (6/6), a specificity of 70% (7/10), and a concordance of 81% (13/16) compared with the matched-tumor-based PCR-CCA (Figure 6a). These results suggest that CCA has the potential to be used as a blood-based clinical diagnostic for ALT+ cancer, although it remains to be investigated whether the low specificity was due to the serum-based CCA providing false positive results or the tumor based PCR-CCA providing false negative results due to the PCR detection step amplifying the double-stranded telomeric repeats as well as the single-stranded CCA products, which can cause false negative PCR-CCA results for the cells with lower C-Circle levels or ALT+ tumor specimens with a substantial healthy (or ALT−) cell component (data not shown).

Based on the ALT+ threshold set by the derivation group’s serum CCA results, 54% (19/35) of the total group was ALT+ (Figure 6a,c), and this data were analyzed with patient and tumor characteristics reported by Dagg et al. [22] (Figure 6a). MYCN amplification is known to be associated with telomerase+ and ALT-negative tumors [22], and 8/9 MYCN amplified cases had ALT-negative serum CCA results, which supports the accuracy of the serum-based CCA whose ALT+ threshold was determined to be blinded to the MYCN status (*p* = 0.039, McNemar test, one-sided). ALT+ was found to be associated with older patients, and the average (SEM) of the ALT+ and ALT− ages were 5.7 (0.5) years and 3.4 (0.6) years, respectively (*p* < 0.01, Mann−Whitney U test, two-sided). There was a non-significant association of ALT+ with female sex (*p* = 0.2, Chi-Squared test, two-sided). These associations require testing in larger independent studies. 

## 4. Discussion

Understanding the process by which C-Circles from ALT+ cancer cells end up in the blood is important for the development of CCA as an ALT-targeted diagnostic. The C-Circle biomarker is a partially single-stranded DNA that is susceptible to blood-based nucleases. Knowing whether C-Circles are secreted in extracellular vesicles that protect them from nucleases is important for understanding C-Circle stability in blood. It was expected that C-Circles would be present in apoptotic bodies that contain nuclear fragments; however, if this were the only source of extracellular C-Circles, then detectable levels of C-Circles in the blood of ALT+ cancer patients may depend on substantial levels of tumor apoptosis. Therefore, it was important to establish if exosomes and/or microvesicles secreted from ALT+ cancer cells contain C-Circles.

We have shown that C-Circles are secreted by ALT+ cancer cells in exosomes. The CCA signal was co-purified with exosomes by differential ultracentrifugation (sedimentation rate), iodixanol gradient separation (density/buoyancy), and immuno-purification (exosomal-specific membrane protein). The C-Circles also appeared to be inside the exosomes, as they were protected from DNAse I digestion that digested all accessible C-Circles. The extracellular media is likely to be of a complex composition and it is not surprising that only 42% of the extracellular C-Circles was found in the exosome-enriched 164K pellet (average over all ALT+ cancer cell lines). C-Circles would also be expected to be released with genomic DNA in apoptotic bodies, and 19% (ALT+ average) of the extracellular C-Circle signal was found in the apoptotic-body-enriched 2K pellet. Only 11% (ALT+ average) of the extracellular C-Circles were found in the microvesicle-enriched 18K pellet, and given the imperfect nature of separation of microvesicles and exosomes by differential centrifugation, there was no evidence for the secretion of C-Circles in the microvesicles. The 18K:164K pellet CCA ratio was 22.5:100, which is similar to the 18K:164K pellet Syntenin ratio of 25:100 (Syntenin is an exosome-specific protein). A substantial proportion of extracellular C-Circles were also found in the 164K supernatant (28% average over all ALT+ cancer cell lines), which could be due to exosomes not sedimented after the 120 min 164K spin and/or free C-Circles released from the necrotic cells. In all of the experiments, >90% of cells were viable; however, with only 0.4% (ALT+ average) of intracellular C-Circles appearing in the extracellular media after the 24 h collection, the contribution from necrotic cells could be significant.

While most (52%) of the CCA signal appeared in the same iodixanol gradient centrifugation fraction (F2) as the exosomes, there was a second broader peak of CCA signal in fractions of a higher iodixanol buoyancy (F4 and F5) that accounted for 20% of the CCA signal. This broader peak suggested that a minority of secreted C-Circles are associated with heterogeneous particles that are not associated with exosome membrane proteins (or microvesicle or apoptotic body proteins), because they did not correspond to Syntenin or Tetraspanin peaks and were from a 164K differential centrifugation pellet that contained minimal Actinin-4 and Calnexin. This minor peak of C-Circles may be associated with protein aggregates, exosome degradation products, or exomeres [44]. 

Although it is not known at what rate C-Circles are created, 0.4% secretion of the total intracellular C-Circles over 24 h seems a low rate, and other catabolic mechanisms may be more important for maintaining a steady state of C-Circle levels in ALT+ cancer cells. The secretion of C-Circles in the exosomes was conserved among the diverse panel of ALT+ cancer cell lines, and therefore secretion may have other advantages for the ALT+ cancer cells. Similar to TERRA [23], C-Circles in the exosomes absorbed by surrounding normal cells may trigger an inflammatory response that supports tumor growth. Silencing of STING1 is common in ALT+ cancer cells in order to prevent the activation of innate immune responses by the elevated level of extrachromosomal telomeric repeats (ECTR) [12,13]. While ECTR, in general, appear to be indirectly associated with ALT, possibly a by-product of the ATRX dysfunction required for ALT activity [11,18], C-Circles appear more tightly associated with the ALT mechanism [3]. Future work to determine if other ECTR and/or TERRA are present in the same exosomes as C-Circles may help understand the mechanism and biological function of C-Circle secretion.

Intracellular C-Circles are tightly linked to ALT activity [3,14], and we have shown here that secreted C-Circles are also a biomarker of ALT activity. All ALT+ cells from a diverse panel of cancer cell lines secreted C-Circles in the exosomes. This panel included cell lines from glioblastoma, non-small cell lung cancer, neuroblastoma, osteosarcoma, and soft tissue sarcoma, as well as neuroblastoma cell lines with low levels of intracellular C-Circles. The findings that C-Circles are actively secreted and protected from nucleases in the exosomes makes the secreted C-Circles robust and stable blood-based biomarkers for ALT+ cancer. Because CCA is an accurate and useful measure of ALT activity in vitro [16], we determined if a blood-based CCA could be an accurate test for ALT+ cancer on serum specimens from 35 high-risk neuroblastoma patients whose ALT status had been determined by CCA (with PCR detection) performed on matching tumor specimens [22]. The serum-based CCA used radioactive dot blot for detection and a CCA level 10-fold that of the healthy control as the threshold for diagnosing ALT activity.

Compared with the tumor-based CCA, the serum-based CCA had a sensitivity, specificity, and concordance of 100%, 70%, and 81%, respectively. Considering that the intra-tumoral heterogeneity (ITH) of ALT has been reported as being as high as 20% [5], ITH in general is known to be high in high risk neuroblastoma, and because the tumor-based CCA was only performed in one site, 100% is an encouraging sensitivity result. The specificity of 70% is unsatisfactory for a diagnostic, and it will need to be determined whether this was due to the serum-based CCA providing false positive results. However, some of these discrepancies may have been due to false negative results from the tumor-based PCR-CCA because the PCR detection step amplifies double-stranded telomeric repeats as well as the single CCA products, and therefore cannot detect C-Circles in the cells (or tissue samples with C-Circles dilute with non-tumor cells) with lower C-Circle levels relative to the telomeric DNA. The serum-based CCA (Figure 6; neuroblastomas) did not have the high levels of non-ALT-specific CCA signal that we found previously in the whole-blood-based CCA (osteosarcomas) [14], which is consistent with the non-ALT-specific signal from whole-blood being present in the blood cells. The isolation of exosomes from the serum by ultracentrifugation was not considered an appropriate method for a potential clinical diagnostic. The serum-based CCA method involved a lysis step before DNA extraction (Materials and Methods), and therefore included C-Circles from the serum exosomes in the extracted DNA.

## 5. Conclusions

In summary, our results demonstrate that a diverse panel of ALT+ cancer cells secreted C-Circles in the exosomes, providing a blood-based biomarker that is both specific for ALT and stable (protected from nucleases). In high-risk neuroblastoma, the blood-based C-Circle assay proved to be a sensitive test for ALT+ cancer, and the apparent low specificity was likely to be due to sensitivity issues of the comparator assay. Both these conclusions need to be investigated with future works; however, the results presented here demonstrate the potential of the blood-based C-Circle assay as a clinical diagnostic for ALT+ cancer, which is required for the development of ALT-targeted therapies as well as for the diagnosis and monitoring of ALT+ cancer.

## Figures and Tables

**Table 1:** Differential centrifugation fraction characteristics; average of SaOS-2 and SK-N-FI.

Characteristic	Percentage of All Pellets Combined; Mean (±95% CI)		
(Protein/DNA)	2K Pellet	18K Pellet	164K Pellet
AB (Calnexin)	84% (9%)	15% (8%)	1% (2%)
MV and AB (Actinin-4)	80% (9%)	19% (7%)	1% (3%)
EXO (Syntenin)	1% (1%)	9% (2%)	90% (1%)
CD9	14% (6%)	38% (3%)	47% (4%)
CD63	20% (3%)	29% (5%)	51% (9%)
CD81	5% (5%)	37% (4%)	57% (7%)
C-Circles	27% (10%)	19% (4%)	53% (10%)

AB—apoptotic bodies; MV—microvesicles; EXO—exosomes.

## Supplementary Materials

The following are available online at https://www.mdpi.com/article/10.3390/cancers13215369/s1. Table S1: Cell lines/strain used in this study, Table S2: Percentage of the total extracellular CCA signal in each fraction, Figure S1: Uncropped Western Blots for Figure 1. Figure S2: Particle size in the 164K differential centrifugation pellets. Figure S3: Overexposed CCA dot blots. Figure S4: Extracellular CCA levels in a panel of ALT+ cancer cell lines. Figure S5: Uncropped Western Blots for Figure 4. Figure S6: Uncropped Western Blots for Figure 5.
