# Supplementary material for: The C-Circle Biomarker Is Secreted by Alternative-Lengthening-of-Telomeres Positive Cancer Cells inside Exosomes and Provides a Blood-Based Diagnostic for ALT Activity
Source: Cancers (Basel). 2021 Oct 26;13(21):5369. doi: 10.3390/cancers13215369 (PMC8582556; doi:10.3390/cancers13215369)
Supplement: Supplementary file 1 [file cancers-13-05369-s001.zip › cancers-1325215-supplementary.pdf]

SUPPLEMENTARY

Table S1: Cell lines/strain used in this study

Cell line/Strain	TMM[1]	Origin	Source
CHLA-90	ALT[2]	Neuroblastoma	Children's Oncology Group cell line repository
DOS16	ALT	Leiomyosarcoma	R D Gupta, Prince of Wales Hospital Sydney, Australia
G-292	ALT	Osteosarcoma	ATCC[4]
SaOS-2	ALT	Osteosarcoma	ATCC
SKLU-1	ALT	Adenocarcinoma, Lung	ATCC
SK-N-FI	ALT	Neuroblastoma	Sigma-Aldrich, Australia
U-2 OS	ALT	Osteosarcoma	ATCC
YTBO	ALT	Glioblastoma	Klaus Holzmann, Medical University Vienna, Austria
ZK-58	ALT	Osteosarcoma	Dr Frans van Valen, Universitätsklinikum Münster, Germany
COG-N-291	ALT	Neuroblastoma	Children's Oncology Group cell line repository
LA-N-6	ALT	Neuroblastoma	Children's Oncology Group cell line repository
A549	TEL[3]	Lung adenocarcinoma	ATCC
HT1080	TEL	Fibrosarcoma	ATCC
MG-63	TEL	Osteosarcoma	Rebecca Mason, University of Sydney, Australia
SH-SY5Y	TEL	Neuroblastoma	Sigma-Aldrich, Australia
SK-N-BE2c	TEL	Neuroblastoma	Sigma-Aldrich, Australia
SJSA-1	TEL	Osteosarcoma	ATCC
TE-85	TEL	Osteosarcoma	ATCC
U-251	TEL	Glioblastoma	Kerry McDonald, University of New South Wales, Australia
HFF5	None	Fibroblast, foreskin	Ralph Böhmer, Ludwig Institute of Cancer Research, Melbourne, Australia.

[1]Telomere maintenance mechanism. [2]Alternative-lengthening-of-telomeres. [3]Telomerase. [4]American Type Culture Collection

Table S2: Percentage of the total extracellular CCA signal in each fraction.

Cell line	Cell number (10^6)*	2K pellet (%)**	18K pellet (%)**	164K pellet (%)**	164K-Supert. (%)**
ZK-58	8.2	14 ± 2	10 ± 2	40 ± 4	36 ± 3
SaOS-2	5.0	24 ± 6	17 ± 2	44 ± 4	15 ± 3
G-292	5.9	20 ± 7	6 ± 2	27 ± 3	47 ± 9
U-2 OS	8.6	19 ± 6	10 ± 3	46 ± 7	26 ± 3
DOS16	2.9	20 ± 4	7 ± 1	52 ± 5	22 ± 2
SK-LU-1	3.5	11 ± 4	8 ± 2	44 ± 4	37 ± 8
CHLA-90	8.6	27 ± 4	10 ± 3	41 ± 8	22 ± 4
SK-N-FI	16.4	17 ± 3	12 ± 1	34 ± 4	38 ± 2
YTBO	2.9	25 ± 7	8 ± 3	39 ± 7	28 ± 4
LA-N-6	19.1	9 ± 3	21 ± 3	51 ± 3	19 ± 3
COG-N-291	15.1	19 ± 2	13 ± 2	49 ± 24	19 ± 1
Average	8.7	18.6	11.1	30.7	28.1

*Number of adherent cells at time of conditioned media harvest
** Percentage of total CCA signal in this differential centrifugation fraction.

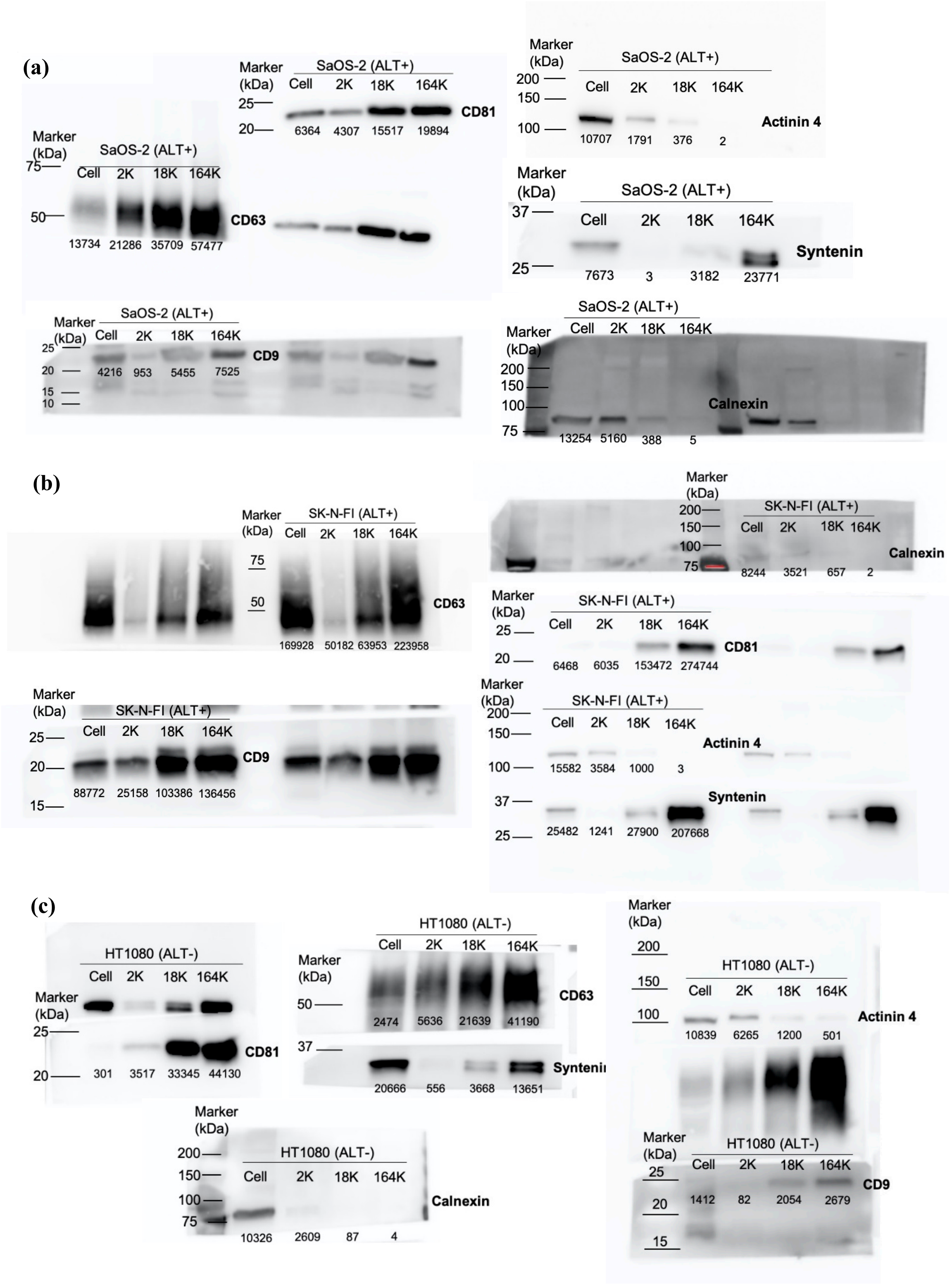

Supplementary Figure S1: Original Western Blot data to show uncropped membrane and densitometry readings of (a) SaOS-2, (b) SK-N-FI and (c) HT1080 in **Figure 1b.** Membranes were cut into smaller strips according to the molecular weight of targeted proteins prior to hybridisation.

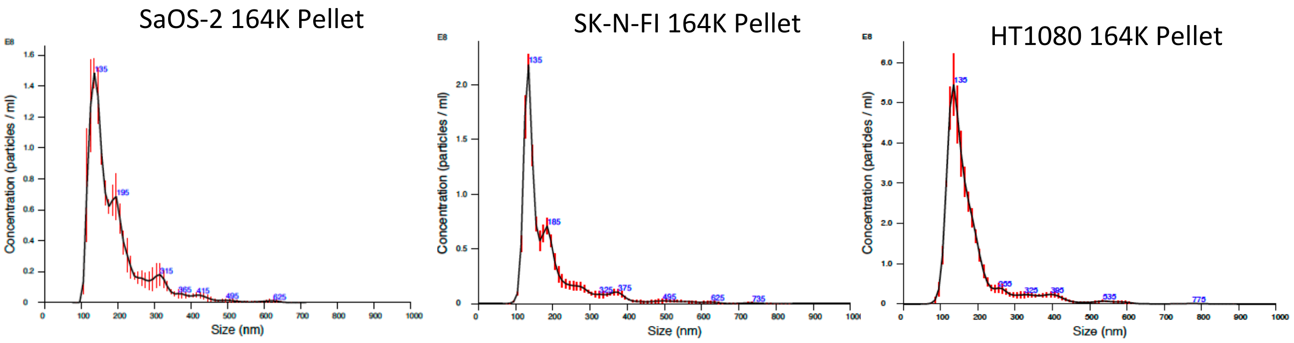

Supplementary Figure S2: Particle size in the 164K differential centrifugation pellets. Nanoparticle tracking analysis (NTA; Nanosight) was performed on 164K differential centrifugation pellets from *SaOS-2*, *SK-N-FI*, and *HT1080*. The histograms are the average from four 60 sec recordings and the red lines show the standard deviations. NTA experiments were not designed to allow comparisons of the absolute number of particles between different cell lines.

This half-page has intentionally been left blank. Please see next page.

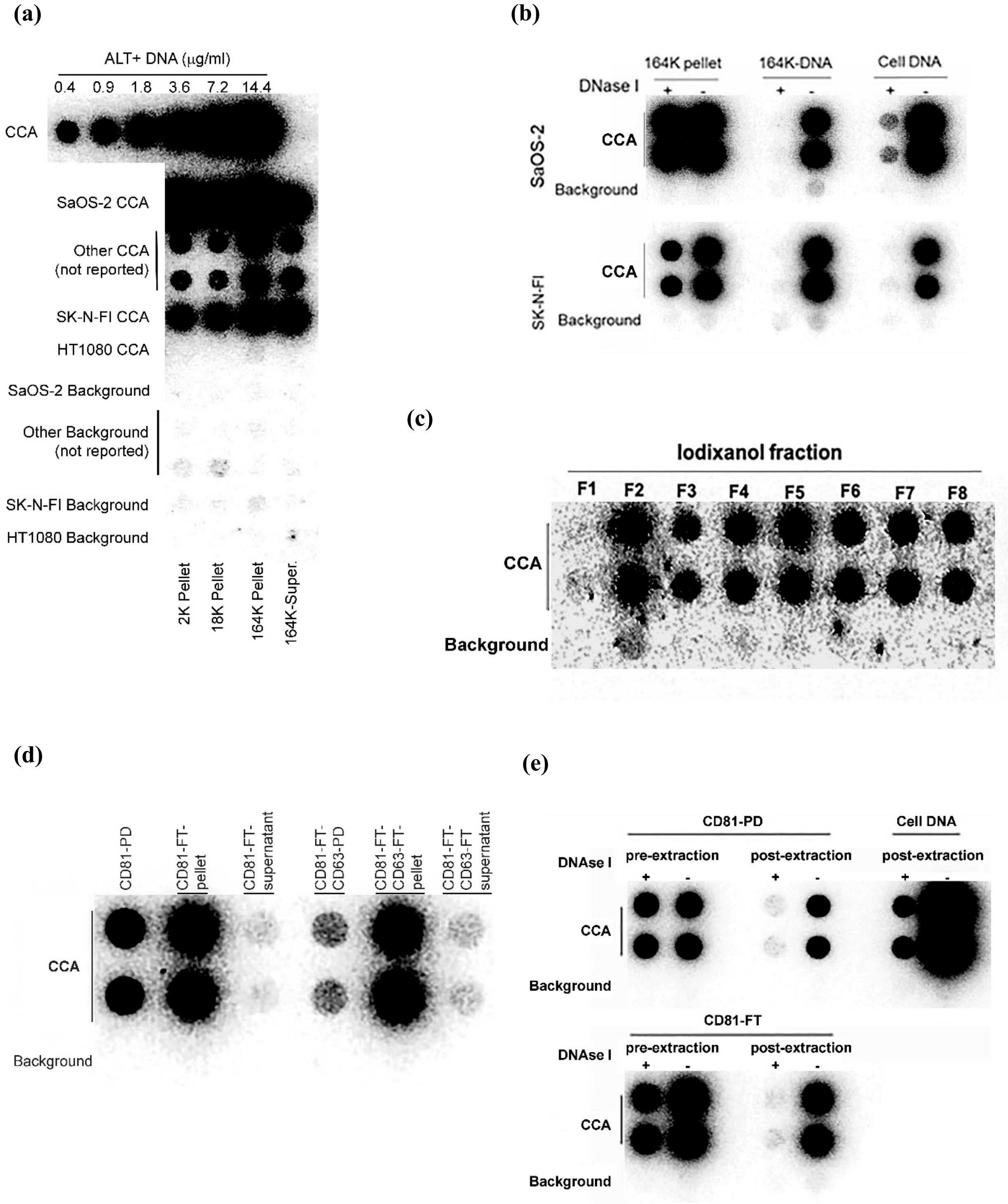

Supplementary Figure S3: Overexposed CCA dot blots to allow better visualization of the *Background* signal in (**a**) **Figure 2a**, (**b**) **Figure 2d**, (**c**) **Figure 4d**, (**d**) **Figure 5b** and (**e**) **Figure 5f** from the main article.

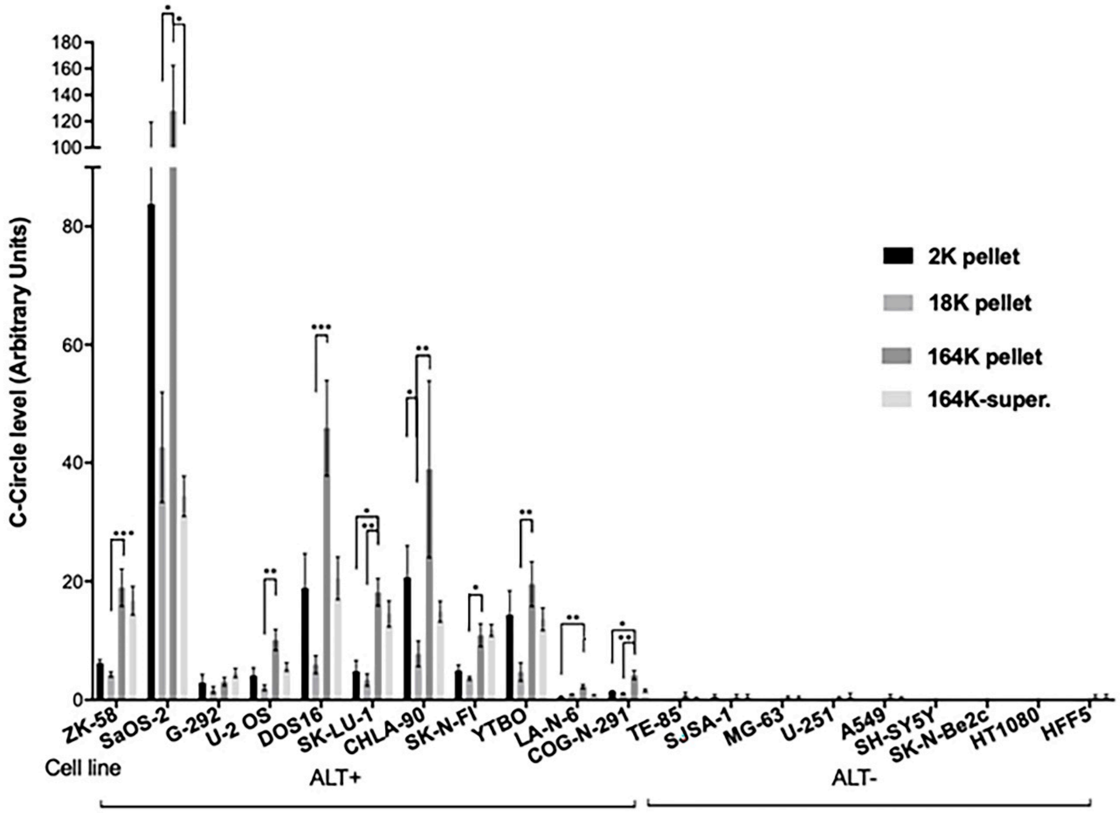

Supplementary Figure S4: Extracellular CCA levels in a panel of ALT+ cancer cell lines. CCA levels for each differential centrifugation fraction of a panel of ALT+ and matched ALT- cancer cell lines and strain (n=3, error bars indicate SEM; * P<0.013, ** P<0.002 and *** P<0.0003 by Friedman test, Bonferroni corrected significance threshold was 0.017 (comparisons of the 164K-pellet to the other fractions only).

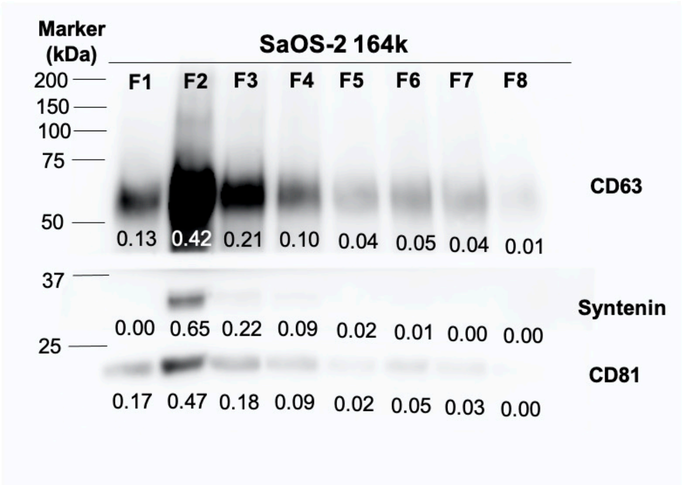

Supplementary Figure S5: Original Western Blot data to show uncropped membrane and intensity ratio of each band in **Figure 4b.** Membranes were cut into smaller strips according to the molecular weight of targeted proteins prior to hybridisation.

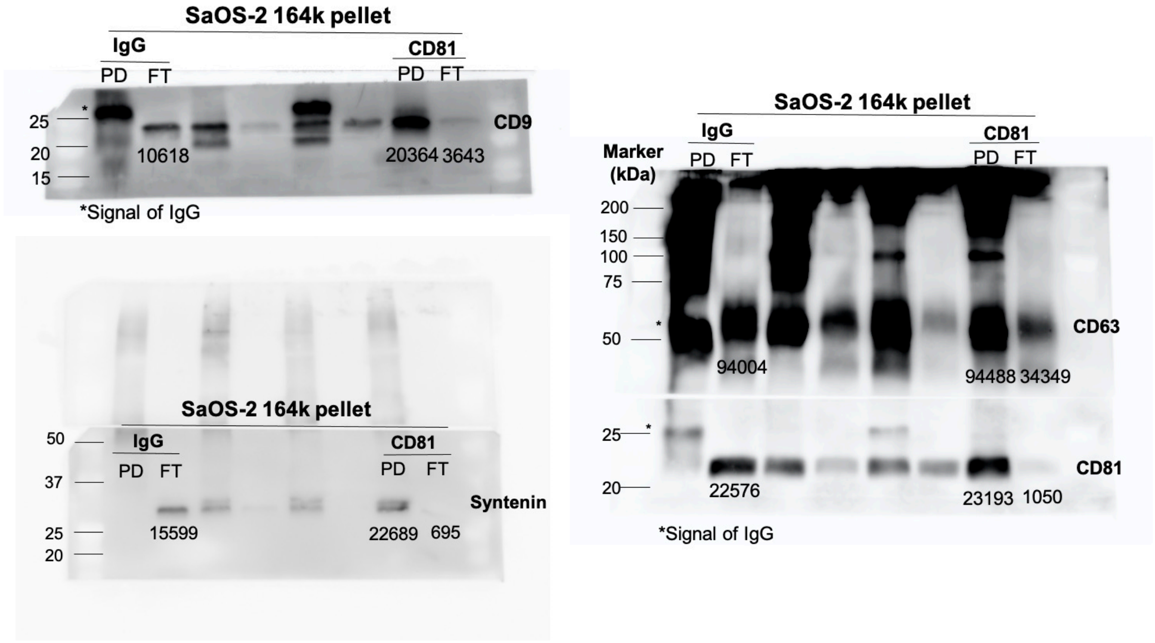

Supplementary Figure S6: Original Western Blot data to show uncropped membrane and densitometry readings of each band in **Figure 5d**. Membranes were cut into smaller strips according to the molecular weight of targeted proteins prior to hybridisation.
